# 3D‐Mixer‐Assisted High‐Entropy Doping of LiNiO_2_ for Co‐Free Ni‐Rich Cathodes in Lithium‐Ion Batteries

**DOI:** 10.1002/advs.76214

**Published:** 2026-06-22

**Authors:** Seung Ri Kim, Thillai Govindaraja Senthamaraikannan, Jun Jae Myeong, Hyung Do Kim, Dong‐Hee Lim, Eun Mi Kim, Sang Mun Jeong

**Affiliations:** ^1^ Department of Chemical Engineering Chungbuk National University Cheongju Chungbuk Republic of Korea; ^2^ Department of Environmental Engineering Chungbuk National University Cheongju Chungbuk Republic of Korea; ^3^ Department of Polymer Chemistry Graduate School of Engineering Kyoto University Katsura Campus, Katsura Kyoto Japan; ^4^ Advanced Energy Research Institute Chungbuk National University Cheongju Chungbuk Republic of Korea

**Keywords:** 3D mixing process, cobalt‐free cathodes, high‐entropy doping, lithium‐ion batteries, Ni‐rich layered cathodes

## Abstract

The development of Co‐free, Ni‐rich cathodes is critical to overcoming cost and sustainability challenges in lithium‐ion batteries. Here, we propose a high‐entropy doping strategy for LiNiO_2_ by uniformly incorporating Mn, Al, Fe, Mg, and Cr into the transition‐metal layer through a scalable 3D mixing process. Each dopant plays a complementary role in stabilizing the layered structure and enhancing electrochemical performance. The resulting Li_1.05_Ni(MnAlFeMgCr)_0.10_O_2_ suppresses Li/Ni cation mixing, strengthens TM─O bonding, mitigates polarization and diffusion bottlenecks, and reduces electrode swelling (from 23% in pristine LNO to 16%). The high‐entropy‐doped cathode delivered a capacity retention of 79% after 100 charge–discharge cycles at 0.3 C, markedly surpassing that of pristine LNO (51%). These results demonstrate that high‐entropy doping effectively improves structural robustness and long‐term durability, offering a practical pathway to economically viable and sustainable next‐generation Co‐free Ni‐rich layered cathodes.

## Graphical abstract

High‐entropy doping of Co‐free Ni‐rich LiNiO_2_ cathodes is achieved through a scalable 3D mixing process. Multi‐element incorporation stabilizes the layered structure, alleviates Li/Ni disorder and electrode swelling, and enhances cycling stability. This approach underscores the potential of high‐entropy strategies to deliver structurally robust and sustainable cathodes for next‐generation lithium‐ion batteries.

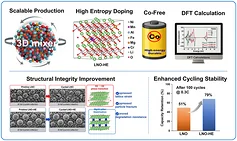

## 1 Introduction

Lithium‐ion batteries (LIBs) are pivotal to the advancement of electric vehicles (EVs), and ongoing innovation is essential to balance the competing demands of high energy density, safety, cost‐effectiveness, and long‐term durability [1, 2, 3, 4]. Among the various components of LIBs, cathode material plays a crucial role in determining their overall performance. In particular, Ni‐rich layered cathode materials such as NCM and NCA have garnered significant interest owing to their high gravimetric/volumetric capacity and well‐established synthesis routes [5, 6, 7]. However, the practical deployment of these cathodes has been hindered by several degradation pathways including Li/Ni cation mixing, oxygen release at high state of charge, lattice collapse during the H2↔H3 transition, surface reconstruction into rock‐salt/spinel phases, and intergranular cracking. These processes contribute to increased polarization and capacity fading [8, 9, 10]. Furthermore, rising cobalt prices and supply chain uncertainties have made the development of Co‐free/less Ni‐rich cathodes a pressing research priority [11, 12]. To address these challenges, several strategies such as elemental doping and surface coating have been explored. Protective layers, such as LiNbO_3_, Li_3_PO_4_, and Al_2_O_3_ reduce electrolyte side reactions, mitigate transition‐metal dissolution, and improve thermal stability [13, 14, 15, 16]. Furthermore, dopants such as Al, Zr, Ti, and Nb have been employed to improve cathode performance [17, 18, 19, 20]. Zhou et al. [13] applied LiNbO_3_ and LiTaO_3_ coatings to the surface of LiCoO_2_ and observed that the LiNbO_3_ coating stabilized the interface by lowering the O 2p states, thereby more effectively suppressing oxygen activity at high delithiation states, whereas the LiTaO_3_ coating facilitated Li^+^ diffusion at the interface owing to smaller Li potential differences. Similarly, Cao et al. [14] demonstrated that a LiNbO_3_ interfacial coating suppressed side reactions and reduced impedance, thereby demonstrating its potential to improve the electrochemical performance of Ni‐rich cathodes. Yin et al. [15] reported that 15 nm Al_2_O_3_‐coated Ni‐rich cathodes (Al_2_O_3_‐LiNi_0.8_Co_0.15_Zn_0.05_O_2_) delivered a reversible capacity of 182 mAh g^−1^ at 0.5C and retained over 94% of the initial capacity after 100 cycles, compared with approximately 67% for uncoated electrodes. This enhancement was attributed to the Al_2_O_3_ protective layer, which suppresses direct contact with the electrolyte, as well as the improved structural stability supported by the increased Li^+^ diffusion coefficient. Collectively, these studies confirm that surface modification via surface coatings can reduce parasitic reactions and stabilize the electrode–electrolyte interface. However, despite these advantages, the protective effect of coating strategies diminishes during prolonged high‐voltage operation, and challenges persist in optimizing the coating thickness and composition [21, 22, 23]. These limitations highlight the need for alternative strategies capable of simultaneously stabilizing both the bulk lattice and electrode–electrolyte interfaces.

Elemental doping strategies have also been extensively investigated to enhance the material properties of battery cathodes. For instance, Al doping reinforces the layered structure and enhances thermal stability; however, it can reduce the Ni‐redox contribution, thereby resulting in capacity loss [17]. Mg doping marginally expands the Li‐slab spacing, facilitating Li^+^ diffusion; however, excessive Mg incorporation can deteriorate electronic conductivity [24]. Ti doping contributes to lattice stabilization but may occupy Li sites, which can in turn increase cation mixing [19]. By contrast, dual/multi‐element doping provide complementary effects. For instance, Al–Zr co‐doping mitigates the influence of the H2–H3 phase transition, whereas Al–Ti co‐doping reduces polarization and improves long‐term cycling stability [25, 26]. Nevertheless, these approaches remain limited, as they are insufficient to comprehensively suppress the multiple degradation mechanisms inherent to Ni‐rich layered oxides.

In this context, high‐entropy oxide (HEO) doping has emerged as a promising approach, utilizing configurational entropy to reduce polarization, alleviate cation disorder, and enhance long‐term electrochemical durability under harsh cycling conditions. By incorporating multiple cations into the transition‐metal layer, HEOs can suppress Li/Ni cation disorder, alleviate the H2–H3 phase transition, and enhance lattice rigidity via increased configurational entropy [27, 28, 29, 30]. Gong et al. [27]. introduced high‐entropy (HE) doping into Ni‐rich cathodes employing multiple dopants such as Mg, Al, Ti, Nb, and Mo. Their results experimentally confirmed that multi‐element doping effectively suppressed structural degradation and improved cycling stability. In particular, suppression of side reactions and reduction of oxygen loss were observed at both the surface and bulk levels. However, the study provided only limited clarification at the electronic‐structure level regarding the specific role of each individual dopant. Li et al. [31]. adopted an HE strategy to suppress oxygen‐induced redox reactions and demonstrated that HE doping modifies the local electronic states, thereby reducing the involvement of (O_2_)^n−^ species (anion redox intermediates). This approach highlights the potential of tuning the electronic structure to suppress excessive oxygen redox activity [32, 33]. However, despite these advances, critical knowledge gaps remain, including the precise role of each cation in stabilizing the entropy‐enhanced lattice, influence of multi‐cation synergy on oxygen redox and Ni migration, and the relationship between electronic structure and observed electrochemical performance. Density functional theory (DFT)‐based studies have provided additional insights into these effects. Bano et al. [34] demonstrated that entropy doping reduces lattice strain and stabilizes Li‐diffusion pathways in Ni‐rich layered oxides, and Ma et al. [35] revealed that the “cocktail effect” of multivalent cations redistributes charge and strengthens structural robustness. However, most previous high‐entropy or multi‐element doping studies have focused on the compositional and electronic‐structure effects of selected dopants, whereas less emphasis has been placed on developing scalable process strategies capable of uniformly introducing multiple dopant species into Co‐free Ni‐rich layered cathodes [27, 28, 32, 35]. In this context, this study differs from conventional single‐, dual‐, and multi‐element doping approaches by integrating a high‐entropy dopant design with a simple, dry‐type, and scalable 3D‐mixer‐assisted homogenization process. This strategy promotes the uniform distribution of equimolar Mn–Al–Fe–Mg–Cr dopants prior to calcination, thereby enabling entropy‐driven lattice stabilization while maintaining practical process scalability for Co‐free LiNiO_2_ cathodes.

This study makes a novel contribution to the literature by demonstrating a scalable HE‐doping strategy for LiNiO_2_, in which equimolar Mn, Al, Fe, Mg, and Cr are incorporated into the transition‐metal layer through a 3D mixing process. Rather than simply utilizing multiple dopant elements, this study introduces a novel approach to integrate high‐entropy compositional design with a practical 3D‐mixer‐assisted route for Co‐free Ni‐rich layered cathodes. The cations play complementary roles: Mn^4+^ suppresses Li/Ni cation mixing, Al^3+^ stabilizes the lattice, Mg^2+^ expands the Li‐slab spacing, Fe^3+^ enhances electronic conductivity, and Cr^3+^ strengthens TM─O bonding. Furthermore, by combining experimental investigations with DFT calculations, we confirmed that the synergistic effects of these cations, in conjunction with configurational entropy stabilization, lead to suppressed polarization, stabilized oxygen redox, enhanced Li^+^ diffusion, and mitigated structural degradation. These results demonstrate that HE design provides a practical and effective strategy to simultaneously achieve high‐voltage stability and long‐term durability in Co‐free Ni‐rich layered cathodes.

## 2 Results and Discussion

**FIGURE 1:**
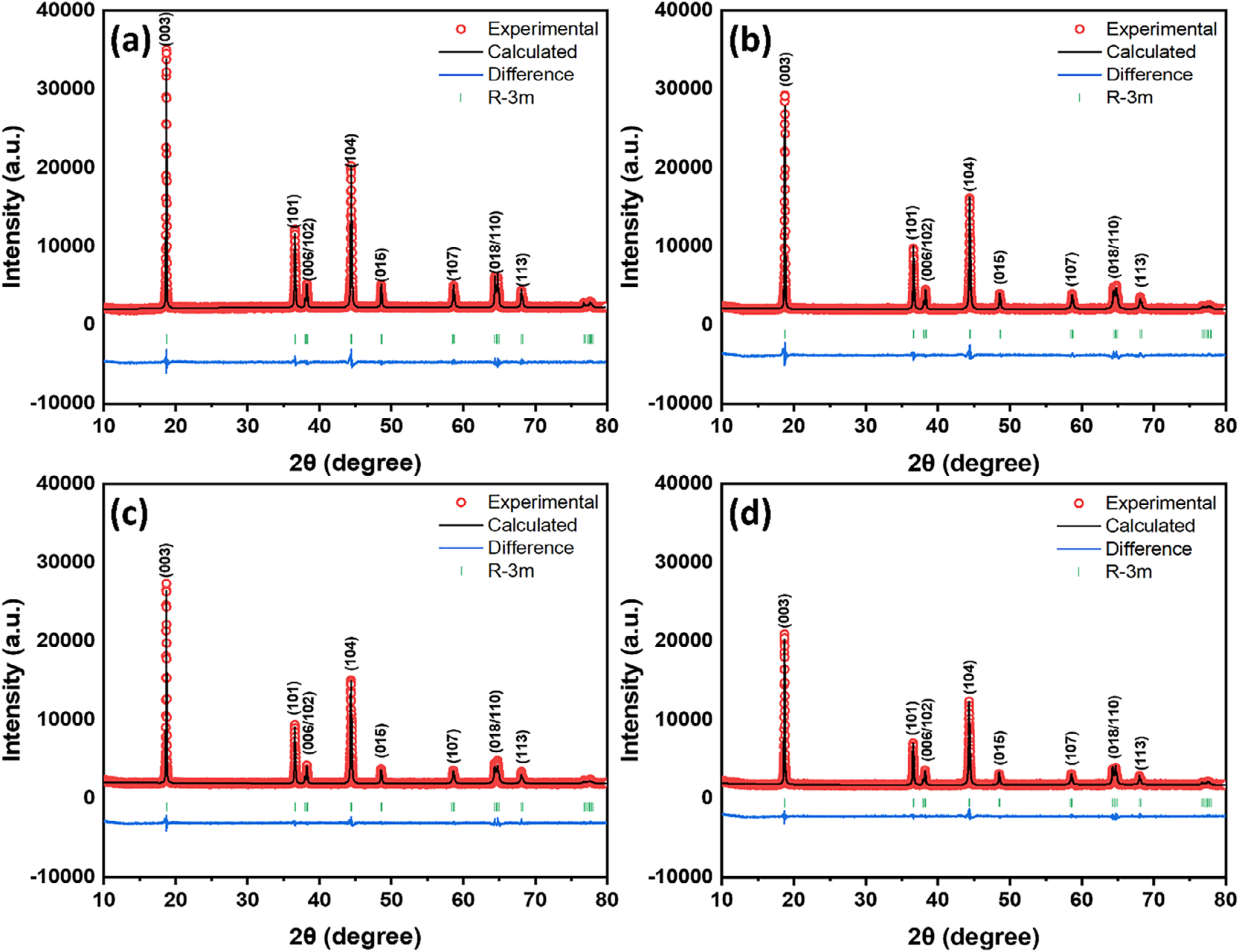
XRD patterns of (a) LNO, (b) LNO‐HE1, (c) LNO‐HE2, and (d) LNO‐HE3 cathode materials.

The α‐3Ni(OH)_2_·2H_2_O precursor synthesized via a hydrothermal process (for a complete description of the materials and methods used in this study, see Experimental Section) exhibited a typical layered hydroxide phase [36], as confirmed by the X‐ray diffraction (XRD) pattern (Figure S1a). Following heat treatment, all powders displayed XRD patterns indexed to a layered rhombohedral structure (*R‐3m*; Figure 1a–d). The HE‐doped LiNiO_2_ (LNO) samples after heat treatment are designated LNO‐HE1, LNO‐HE2, and LNO‐HE3, corresponding to the doping concentrations of 0.05, 0.10, and 0.15 mol, respectively. Rietveld refinements yielded low residuals (*R_p_* ≈ 2.60–2.74%, *R_wp_* ≈ 3.48–5.51%) and randomly distributed difference curves, validating the adequacy of a single‐phase model (Table S1). The refined atomic coordinates and site occupancies are summarized in Table S1. The *a* and *c* lattice parameters and unit cell volume expanded marginally with increasing dopant concentration (*a*: 2.875 → 2.879 Å; *c*: 14.193 → 14.226 Å; *V*: 101.63 → 102.14 Å^3^). The modest increase in the *c/a* ratio (≈4.936 → ≈4.940) suggests an expansion of the Li slab spacing and improved layered ordering [37]. This trend is attributed to the suppression of Ni/Li antisite disorder by the strong M–O bonding and charge‐compensation effects introduced by the HE dopants (Mn, Al, Fe, Mg, Cr) within the transition‐metal layers [38, 39]. The intensity ratio *I_(003)_/I_(104)_* exceeded 1.4 for all samples and was typically higher in the doped specimens than in the undoped counterpart, with the values for LNO, LNO‐HE1, LNO‐HE2, and LNO‐HE3 being 1.461, 1.877, 1.881, and 1.767, respectively (Table S1). LNO‐HE2 exhibited the highest *I_(003)_/I_(104)_* ratio, indicating that the optimized HE dopant concentration is most effective in mitigating Li/Ni intermixing while enhancing layered ordering [40]. Clear splitting of the *I_(006)_/I_(102)_* and *I_(018)_/I_(110)_* doublets further corroborated the preservation of *R‐3m* symmetry and robust layered ordering [41, 42].

**FIGURE 2:**
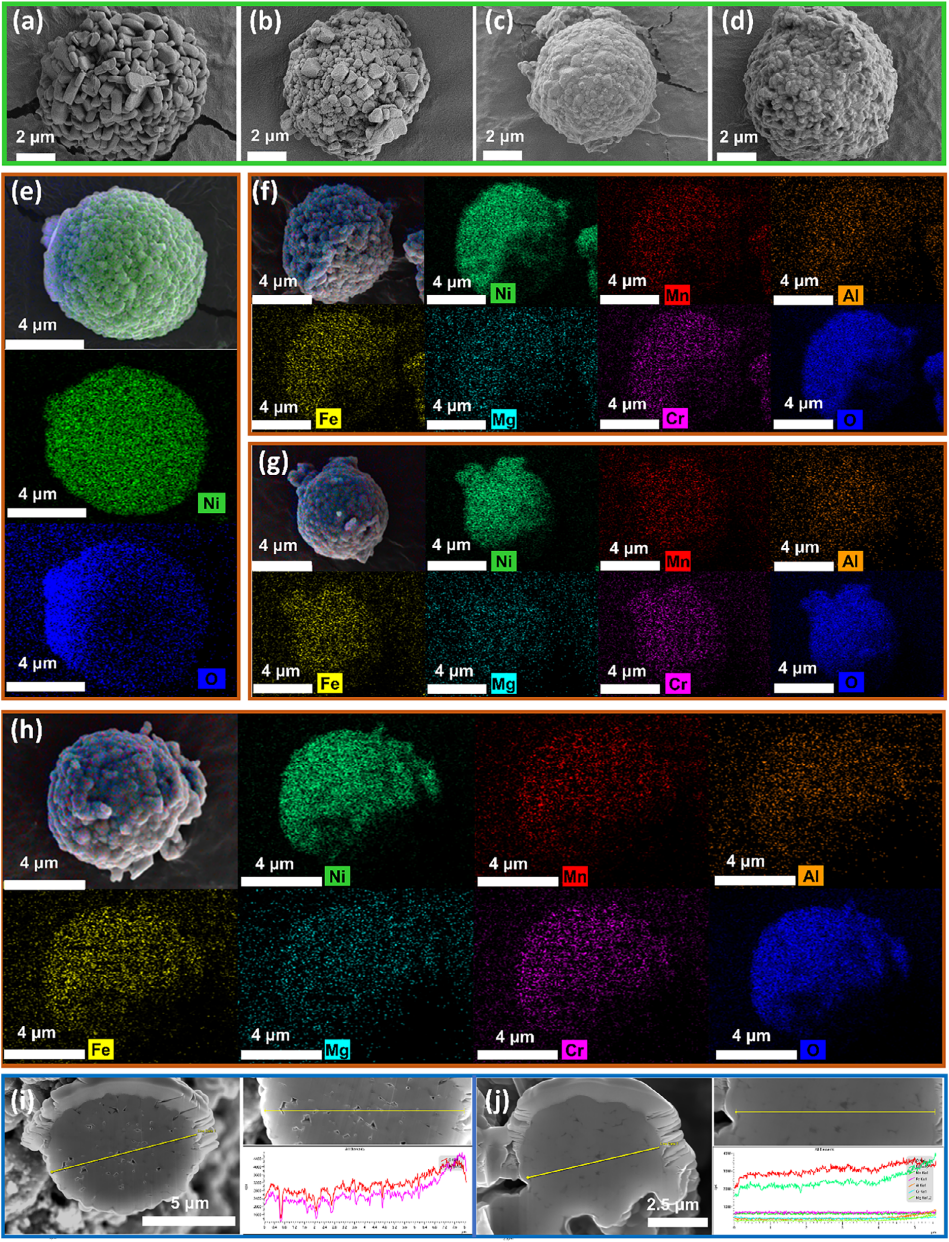
FE‐SEM and EDS mapping images showing the morphology and elemental distribution in the LNO and LNO‐HE cathode materials. FE‐SEM images of the secondary particles for (a) LNO, (b) LNO‐HE1, (c) LNO‐HE2, and (d) LNO‐HE3. EDS elemental mapping images of (e) LNO, (f) LNO‐HE1, (g) LNO‐HE2, and (h) LNO‐HE3, demonstrating the distribution of Ni, O, and the dopant elements. (i, j) Cross‐sectional SEM images of the FIB‐milled LNO and LNO‐HE2 particles and their corresponding EDS line‐scan profiles, respectively.

Scanning electron microscopy (SEM) was employed to investigate the microstructure and morphology of the hydrothermally synthesized precursor and the calcined powders. SEM observations revealed that the precursor particles possessed a spherical secondary morphology with a diameter of approximately 10 µm, estimated from 30 randomly selected particles, and comprised fine, thread‐like primary particles entangled to form a porous structure (Figure S1b). Energy‐dispersive spectroscopy (EDS) mapping further demonstrated that the constituent elements were uniformly distributed throughout the spherical precursor particle, confirming the homogeneous chemical composition of the precursor (Figure S1c). Such morphological and compositional uniformity in the precursor is critical for maintaining the structural integrity during subsequent calcination. Following calcination, the powders transformed into layered rhombohedral oxides while preserving the spherical morphology of the secondary particles (Figure 2). The calcined powders comprised primary particles with an average size of 500–800 nm (estimated from 30 randomly selected primary particles in the SEM images). These primary particles were agglomerated or sintered into spherical secondary particles of approximately 10 µm in diameter. The undoped LNO sample formed irregular aggregates containing numerous open surface pores and fragmented plate‐like primary grains (Figure 2a). By contrast, the HE‐doped samples exhibited progressively improved sphericity and surface densification with increasing doping concentration, and LNO‐HE2 showed the smoothest surface and most uniform morphology (Figure 2b–d). This optimized microstructure is expected to reduce the interfacial area available for parasitic reactions with the electrolyte and mitigate the stress concentration at particle boundaries during cycling [43, 44]. However, in LNO‐HE3, excessive doping resulted in renewed surface roughness, protruding sub‐particles, and local open pores, which may promote electrolyte penetration and crack propagation during cycling [45].

Importantly, this concentration‐dependent morphological trend correlates with the XRD/Rietveld refinement results. As the HE dopant concentration increased from LNO‐HE1 to LNO‐HE2, the secondary particles exhibited enhanced sphericity, surface densification, and morphological uniformity relative to pristine LNO. Specifically, LNO‐HE2 exhibited the most uniform and compact secondary‐particle morphology, which aligns with its favorable Rietveld parameters and maximum *I_(003)_/I_(104)_* ratio. These results indicate that an optimized HE dopant concentration promotes both lattice stabilization and secondary‐particle densification. In particular, the denser and more uniform secondary particles of LNO‐HE2 are expected to suppress electrolyte penetration during cycling and alleviate local stress concentrations, thereby enhancing structural stability. However, the further increase in dopant concentration in LNO‐HE3 led to a slight decrease in *I_(003)_/I_(104)_* and the reappearance of surface roughness and local open pores. This suggests that excessive HE doping may partially disturb the optimized layered ordering and particle growth behavior. Therefore, the HE dopant concentration must be carefully controlled to balance lattice stabilization, the suppression of Li/Ni antisite disorder, and secondary‐particle morphology.

EDS mapping confirmed that Ni, Mn, Al, Fe, Mg, and Cr were uniformly distributed across the secondary particles of the HE‐doped samples (Figure 2e–h). However, because surface elemental mapping alone is insufficient to verify whether the dopant elements are incorporated into the bulk region or merely segregated near the particle surface, cross‐sectional SEM and EDS line‐scan analyses were performed on focused ion beam (FIB)‐milled LNO and LNO‐HE2 particles (Figure 2i,j). The line‐scan profile for the undoped LNO particle exhibited only Ni and O signals across the particle cross‐section, as expected. In contrast, the line‐scan profile for the FIB‐milled LNO‐HE2 particle exhibited continuous Mn, Al, Fe, Mg, and Cr signals together with Ni and O signals throughout the particle cross‐section. These dopant signals were not confined to the outer surface region but were detected across the cross‐sectional diameter of the secondary particle. These results indicate that the HE dopant elements are homogeneously distributed within the bulk secondary particles rather than being localized as surface residues or segregated phases. Although EDS analysis alone cannot directly determine the exact crystallographic site occupancy of each dopant element, the combination of the homogeneous cross‐sectional elemental distribution and the absence of detectable secondary phases in the XRD/Rietveld refinement results strongly supports the successful incorporation of Mn, Al, Fe, Mg, and Cr into the LiNiO_2_‐based layered oxide structure.

**FIGURE 3:**
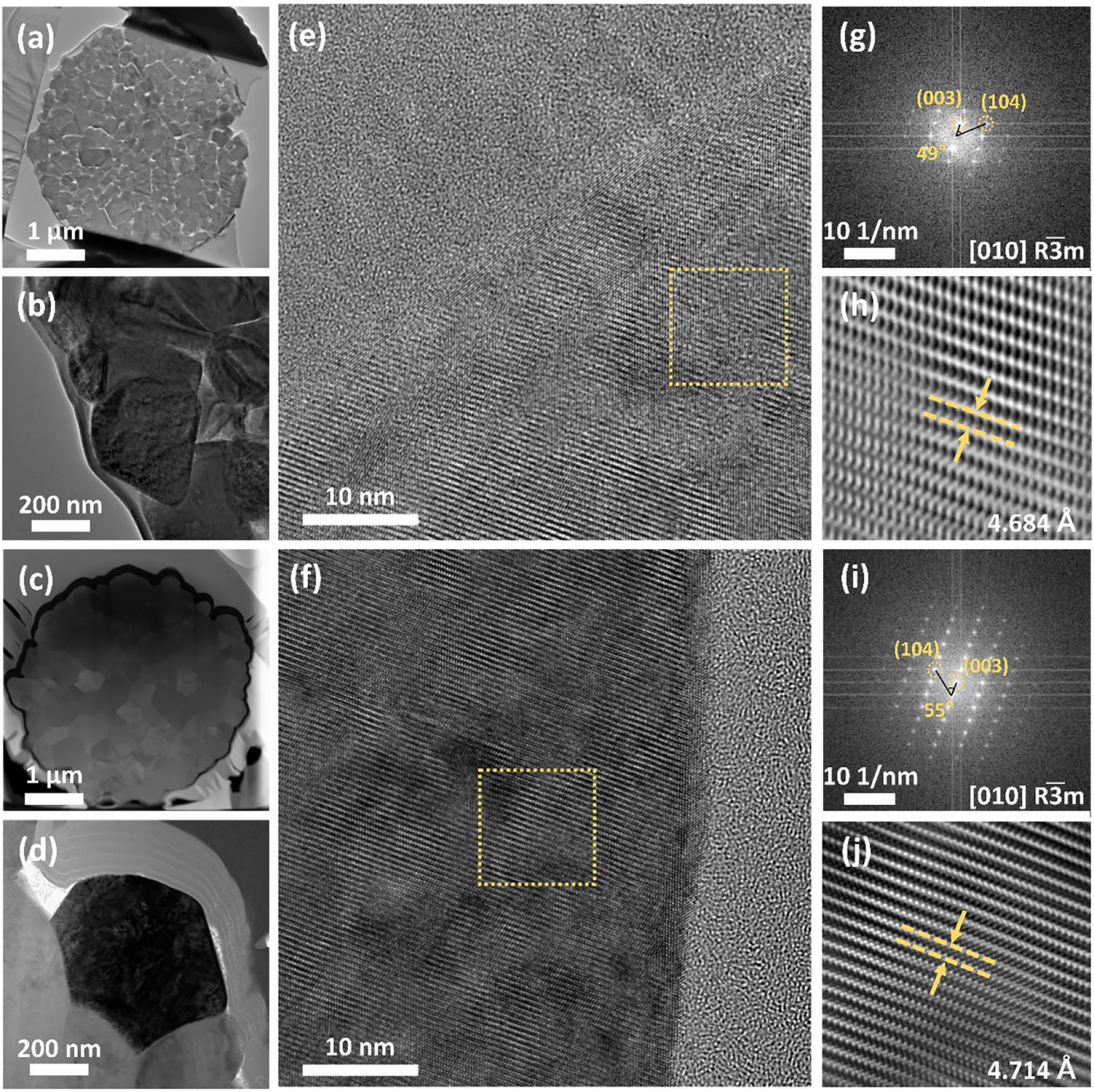
HR‐TEM images of (a, b, e, g, h) LNO and (c, d, f, i, j) LNO‐HE2 cathode materials. (a, c) Low‐magnification TEM images of secondary particles. (b, d) High‐magnification images showing primary particle boundaries. (e, f) HR‐TEM image of the particle edge. (g, i) Fast Fourier transform (FFT) pattern corresponding to the boxed area. (h, j) Lattice fringe image indicating the *d*‐spacing corresponding to the (003) plane.

Transmission electron microscopy (TEM) analysis revealed that the undoped LNO had numerous discernible primary‐particle boundaries and local pores within fragmented secondary‐particle pieces (Figure 3a, b). By contrast, the HE‐doped sample (LNO‐HE2) exhibited uniform contrast and smooth particle contours, indicating a more densely consolidated interior (Figure 3c, d). These microstructural differences are expected to mitigate electrolyte penetration and alleviate stress concentration [46]. At higher magnification, using high‐resolution TEM (HR‐TEM), continuous lattice fringes can be observed extending to the particle surface in both LNO and the LNO‐HE2 specimen (Figure 3e, f). The measured spacings of the (003) fringes were *d*_(003)_ ≈ 4.684 Å for LNO and 4.714 Å for LNO‐HE2, agreeing within 1% with the XRD‐derived Li‐slab spacings (LNO: *c* ≈ 14.1929 Å → *c*/3 ≈ 4.7310 Å; LNO‐HE2: *c* ≈ 14.2255 Å → *c*/3 ≈ 4.7418 Å). The corresponding fast Fourier transform (FFT) patterns acquired along the (010) zone axis clearly resolved the (003) and (104) reflections with inter‐reflection angles (49° for LNO and 55° for LNO‐HE2) and spot symmetry consistent with the layered rhombohedral (*R‐3m*) structure (Figure 3g–j).

**FIGURE 4:**
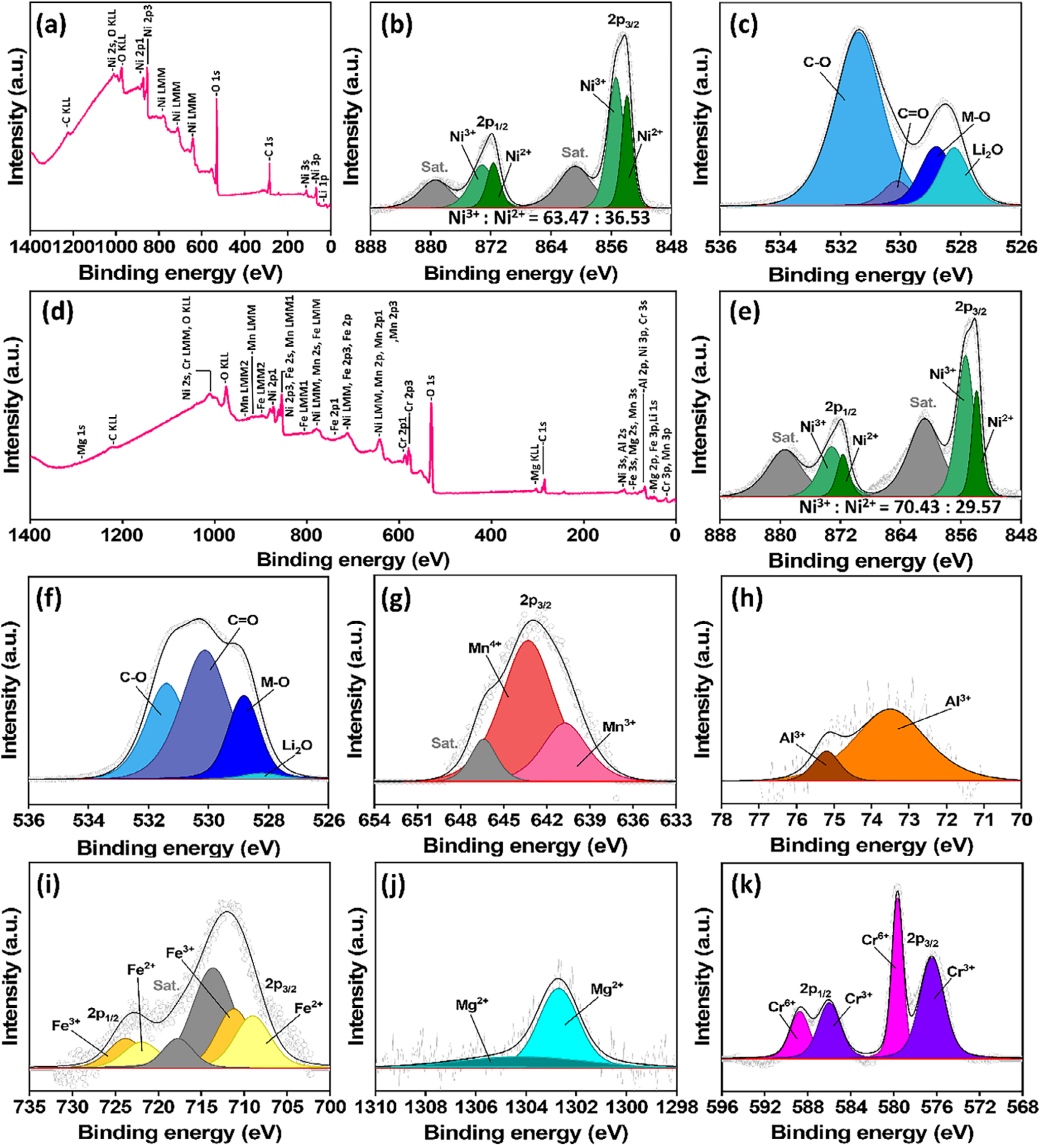
XPS spectra of (a–c) LNO and (d–k) LNO‐HE2 powders. (a, d) Survey spectra, (b, e) Ni 2p, (c, f) O 1s, (g) Mn 2p, (h) Al 2p, (i) Fe 2p, (j) Mg 1s, (k) Cr 2p.

The X‐ray photoelectron spectroscopy (XPS) spectra of pristine LNO and LNO‐HE2 (Figure 4) provide further insights into the surface chemistry and oxidation states of the constituent elements. The survey spectrum confirms that both samples contain the expected Ni, O, C, with no discernible impurity peaks (Figure 4a, d). In contrast to LNO, the doped sample additionally exhibits distinct peaks corresponding to Mn, Al, Fe, Mg, and Cr, verifying the successful incorporation of the HE dopants into the lattice without introducing secondary phases. In the Ni 2p region, both LNO and the doped sample show Ni^2+^ and Ni^3+^ contributions at 853.9 and 855.4 eV, respectively [47]. However, the doped sample displays a higher Ni^3+^/Ni^2+^ ratio (70.43: 29.57) compared with that of LNO (63.47: 36.53). This increase in Ni^3+^ fraction indicates a higher average Ni valence and reduced proportion of Ni^2+^, which is more prone to migrate into Li layers [48]. Such a shift aligns with the XRD observations of an increased *I_(003)_/I_(104)_* intensity ratio and expansion of the c/3 parameter, both indicative of suppressed Li/Ni cation mixing and enhanced layered ordering relative to pristine LNO (Figure 4b, e) [49]. The O 1s spectra further support these findings. LNO exhibits a larger fraction of Li_2_O–related non‐lattice oxygen, whereas the doped sample shows a dominant lattice oxygen (M─O) contribution and a substantially reduced Li_2_O signal [30, 50]. This reduction of non‐lattice oxygen species suggests that HE doping stabilizes the oxygen framework, leading to a more robust surface chemistry and mitigating oxygen loss during cycling [51] (Figure 4c, f). The additional dopants in the LNO‐HE2 sample are identified as Mn^4+^ (Mn 2p_3/2_, 643.29 eV), Al^3+^ (Al 2p, 73.5 eV), Fe^3+^ (Fe 2p_3/2_, 711.20 eV), Mg^2+^ (Mg 1s, 1302.70 eV), and Cr^3+^ (Cr 2p_3/2_, 576.44 eV) (Figure 4g–k). Their stable oxidation states confirmed that these dopants were incorporated into the transition‐metal layers without forming undesired reduced or oxidized species [52, 53, 54, 55, 56]. Notably, such multi‐element incorporation enhances M─O bonding strength and suppresses surface defect formation [51, 57], both of which are absent in pristine LNO. Overall, compared with pristine LNO, the HE‐doped composition demonstrated not only an increase in average Ni valence and stronger lattice oxygen bonding but also stable incorporation of multiple dopant cations. These features collectively contribute to reduced surface defects, improved interfacial robustness, and enhanced electrochemical stability during prolonged cycling [58].

**FIGURE 5:**
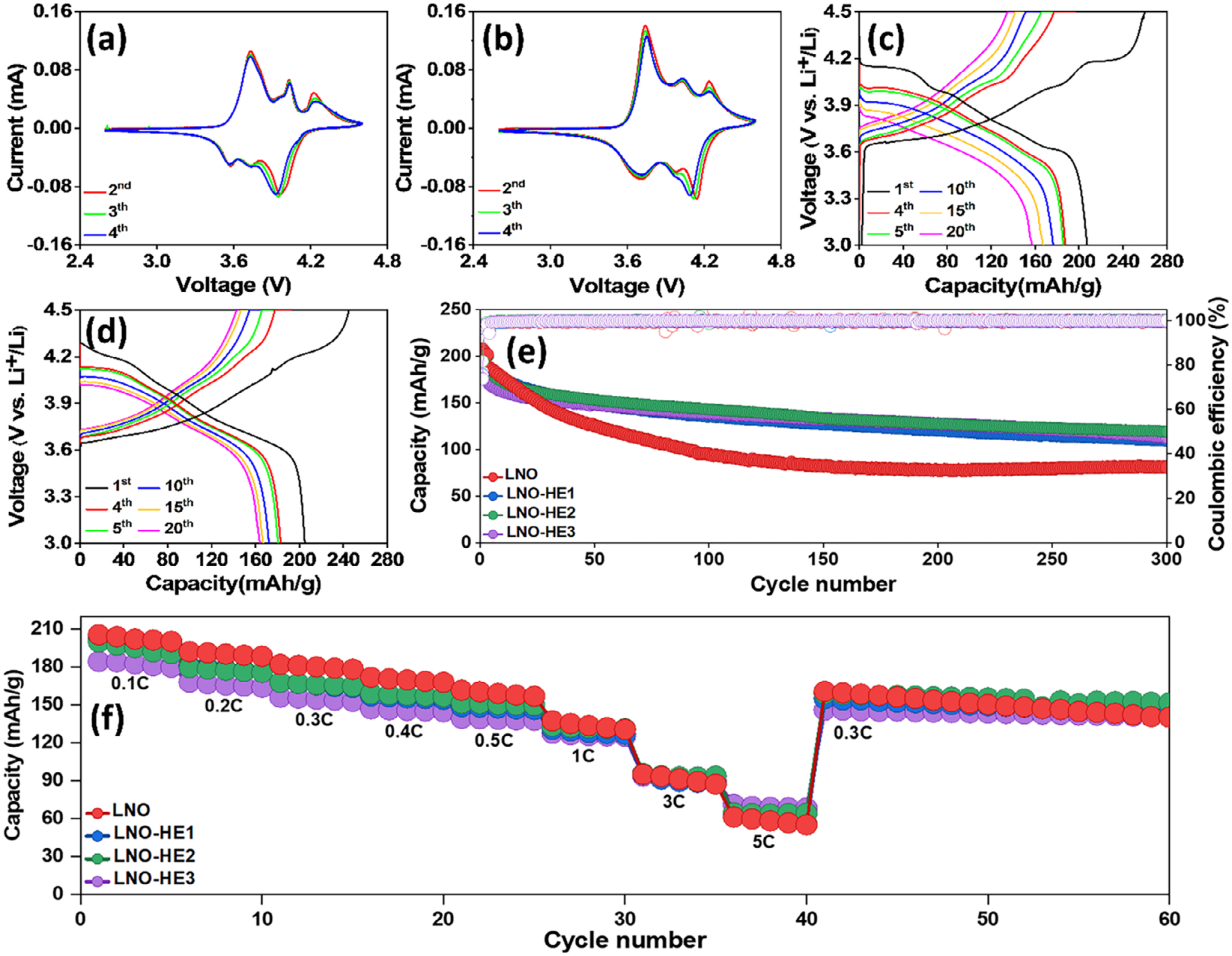
Electrochemical performance of the LNO and LNO‐HE cathode materials. CV curves of (a) LNO and (b) LNO‐HE2 measured in the voltage range of 2.6–4.6 V versus Li^+^/Li at a scan rate of 0.05 mV s^−1^. GCD curves at selected cycles for (c) LNO and (d) LNO‐HE2. (e) Cycling performance and Coulombic efficiency at 0.3C over 300 cycles. To stabilize the electrodes, the cycling performance was assessed at 0.1C for the initial three formation cycles, followed by cycling at 0.3 C. (f) Rate capability at current rates ranging from 0.1C to 5C.

The electrochemical characteristics of the LNO and LNO‐HE cathodes are shown in Figure 5. LNO exhibits distinct redox peak associated with the H1 →M → H2 → H3 phase transitions within the voltage window of 2.6–4.6 V (Figure 5a). However, these peaks are relatively broad and asymmetric, with a significant voltage separation between oxidation and reduction, indicating severe polarization and structural instability [59, 60]. LNO‐HE1 and LNO‐HE3 (Figure S2a, b) initially show cyclic voltammetry (CV) peaks, but these peaks gradually shift and weaken upon cycling. This behavior suggests that increased surface roughness and local structural heterogeneity have been induced, leading to deteriorated electrochemical stability [61]. By contrast, LNO‐HE2 (Figure 5b) displays sharper and more symmetric redox peaks with a smaller voltage gap, indicating enhanced reversibility and suppressed polarization [62]. Notably, the stabilization of the H2 ↔ H3 transition indicates that HE doping effectively mitigates structural degradation at high voltage operation [27].

The galvanostatic charge–discharge (GCD) profiles further validate these observations. For LNO (Figure 5c), pronounced voltage hysteresis and progressive polarization growth are observed with cycling, in conjunction with significant changes in the plateau regions, which can be attributed to increasing Li/Ni cation disorder and accumulated structural deterioration [63]. By contrast, the LNO‐HE sample (Figure 5d; Figure S2c, d) maintains stable GCD profiles with significantly suppressed polarization growth, particularly in the H2 ↔ H3 region, clearly demonstrating the structural stabilization effect of HE doping [64]. These results collectively confirmed that LNO‐HE2 achieved the most optimized electrochemical stability and structural durability among the investigated compositions.

In the galvanostatic intermittent titration technique (GITT) profiles (Figure S2e, f), the Li^+^ diffusion coefficients (D_Li_^+^) of all samples are on the order of 10^−8^ cm^2^ s^−1^ in the charging voltage range of 3.6–4.1 V. However, in the voltage range of 4.1–4.3 V (corresponding to the H2↔H3 transition), LNO shows the sharpest drop to the lowest D_Li_^+^ values and largest fluctuation range, indicating a severe diffusion bottleneck across the phase‐transition region [65]. By contrast, the HE‐doped samples display smaller depressions and flatter traces in this window; notably, the LNO‐HE2 sample maintains the highest minimum D_Li_^+^ value, indicating the most effective mitigation of diffusional degradation. On discharge, the LNO‐HE2 sample sustains a relatively higher D_Li_^+^ value across the entire voltage window and exhibits faster recovery. The LNO‐HE1 and LNO‐HE3 electrodes show intermediate improvements, while LNO exhibits delayed recovery of D_Li_^+^ at high voltages owing to hysteresis [66] and a generally lower overall diffusivity level.

Figure 5e shows the cycling performance of each cathode. LNO delivered an initial discharge capacity of 207.4 mAh g^−1^ at 0.1 C, but this value dropped to < 180 mAh g^−1^ by the fourth cycle (0.3 C). Continued cycling resulted in rapid capacity fading, with only 51% capacity retention after 100 cycles. By contrast, all LNO‐HE electrodes outperformed LNO in terms of retention; notably, the LNO‐HE2 composition maintained 79% of its capacity after 100 cycles. This performance improvement is attributed to configurational‐entropy‐driven lattice stabilization, which alleviates voltage heterogeneity and reduces stacking‐fault density, a marginal expansion of the Li‐slab spacing that improves Li^+^ diffusion properties, and a densified surface that suppresses parasitic electrolyte reactions and transition‐metal dissolution [57].

The rate capability results (Figure 5f) show that the HE‐doped samples, particularly LNO‐HE2, deliver higher discharge capacities and better retention at both low and high rates, with the smallest capacity loss upon returning to the low rate (3.6%). By contrast, LNO exhibits a higher initial capacity but experiences a pronounced capacity drop at elevated rates, in conjunction with the highest recovery loss (9.8%). LNO‐HE1 composition also retains high capacity at a low rate but declines progressively with increasing rate, exhibiting a recovery loss of 5.4%. LNO‐HE3 maintains relatively stable capacity retention across rates and a low recovery loss (4.6%); however, its absolute capacity is reduced from the outset, consistent with diminished Ni‐redox activity caused by over‐doping [67]. The rate‐dependent GCD profiles further support these trends (Figure S3a–d). All electrodes exhibited shortened charge/discharge plateaus and increased voltage polarization with an increasing current rate from 0.1 to 5C. However, the LNO electrode exhibited a more pronounced polarization increase and severe capacity loss at high rates (Figure S3a), indicating sluggish reaction kinetics and larger kinetic limitations [12]. Conversely, LNO‐HE2 maintained more distinct charge/discharge profiles with reduced polarization even at elevated rates (Figure S3c), suggesting improved Li^+^ transport kinetics and enhanced electrochemical reversibility. LNO‐HE1 exhibited an intermediate performance (Figure S3b), whereas LNO‐HE3 displayed a relatively stable profile retention but lower overall capacity (Figure S3d), consistent with the rate capability results. These GCD profiles confirm that the optimized HE doping in LNO‐HE2 enhances the high‐rate charge/discharge performance while maintaining a favorable balance between structural stabilization and redox activity.

Table S2 provides a comparative summary to contextualize the electrochemical performance of LNO‐HE2 among recently reported Co‐free Ni‐rich layered cathodes. This comparison evaluates representative Co‐free Ni‐rich cathode materials in terms of discharge capacity and capacity retention under their respective cycling conditions. Although direct comparison is constrained by voltage range, current density, electrolyte composition, and testing protocol variations, LNO‐HE2 exhibits a competitive cycling stability while maintaining a highly Ni‐rich and Co‐free composition. Specifically, the improved capacity retention of LNO‐HE2 aligns with the suppressed secondary‐particle collapse, mitigated impedance growth, and enhanced structural reversibility confirmed by postmortem SEM, in situ EIS, and in situ/ex situ XRD analyses. These results indicate that the 3D‐mixer‐assisted HE‐doping strategy provides a practical and effective route for enhancing the durability of Co‐free Ni‐rich layered cathodes.

**FIGURE 6:**
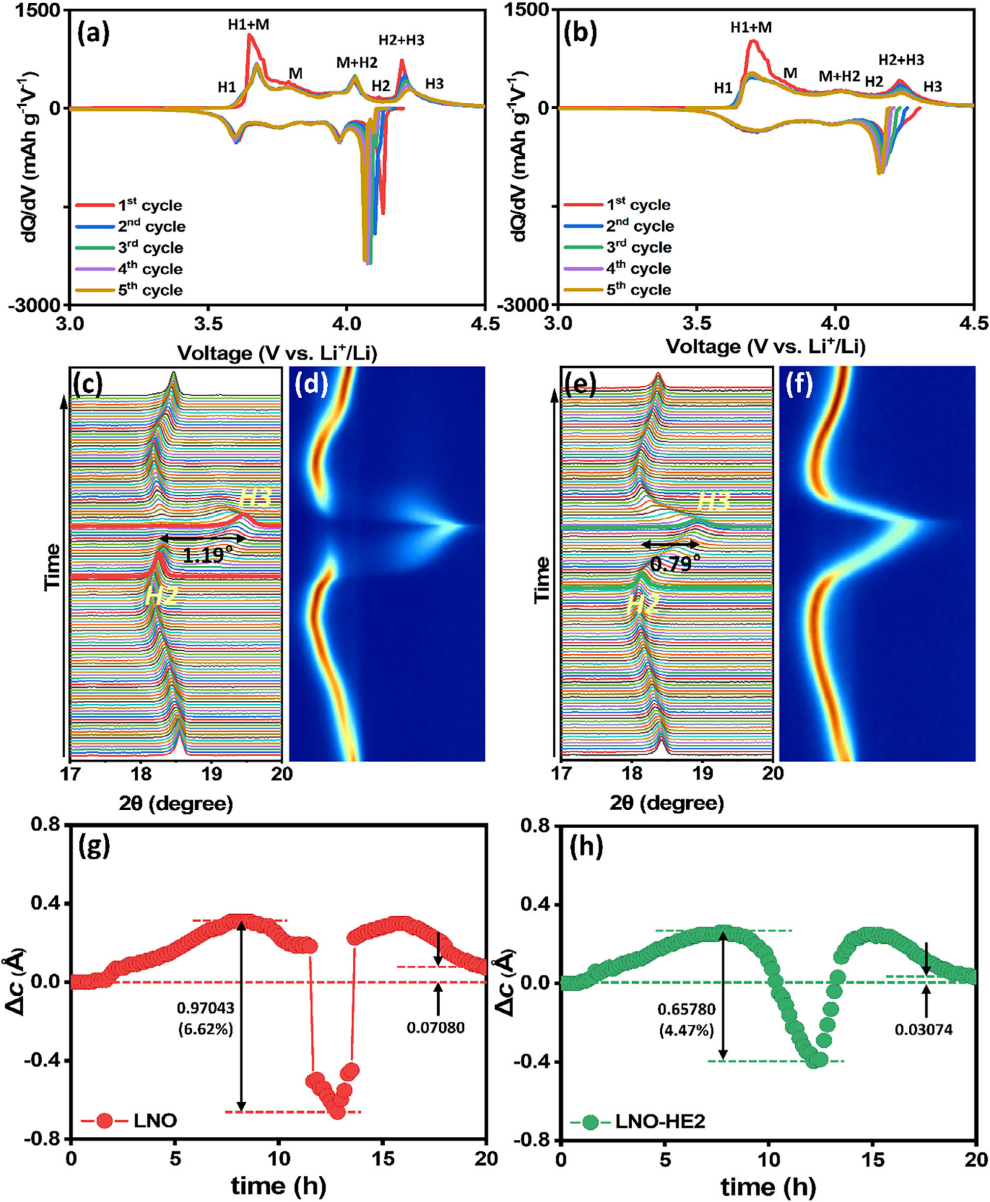
Differential capacity (dQ/dV) plots of (a) LNO and (b) LNO‐HE2 for different cycles measured at 0.1C. In situ XRD patterns of (c, d) LNO and (e, f) LNO‐HE2 collected during electrochemical cycling between 3.0 and 4.5 V at 0.1C. (g, h) Evolution of the c lattice parameters of LNO and LNO‐HE2 calculated from the (003) peak positions.

The differential capacity (dQ/dV) profiles of LNO and LNO‐HE2 recorded over the initial cycles (first–5^th^) are shown in Figures 6a,b, respectively. LNO (Figure 6a) exhibits well‐resolved H1–M–H2–H3 features, including a pronounced, sharp peak in the H2↔H3 transition region [68]. In contrast, LNO‐HE2 (Figure 6b) shows visibly broadened features with reduced peak amplitudes, and the H2+H3‐related features at the high voltage appear less distinct over the same cycles [69, 70]. Figure 6c–f presents the in situ XRD patterns focusing on the (003) reflection during electrochemical cycling. For both electrodes, the (003) peak shifts toward lower angles in the intermediate‐voltage region and toward higher angles in the high‐voltage region. Notably, the total (003) peak shift for LNO‐HE2 across the H2–H3 transition region is smaller (0.79°; Figure 6e) than that for LNO (1.19°; Figure 6c). The c‐axis lattice‐parameter change (*Δc*) derived from the (003) peak positions further highlights this peak shift difference: LNO exhibits a maximum *Δc* of 0.97043 Å (6.62%) with a residual (non‐recovered) *Δc* of 0.07080 Å after discharge (Figure 6g), while LNO‐HE2 shows a smaller maximum *Δc* of 0.65780 Å (4.47%) and a reduced residual component of 0.03074 Å (Figure 6h). Collectively, LNO‐HE2 displays a smaller high‐voltage (003) peak shift and reduced magnitude and residual component of the c‐axis change compared with LNO. These results suggest that HE doping mitigates c‐axis contraction and enhances structural reversibility during charge/discharge, thereby alleviating high‐voltage instabilities associated with the H2→H3 transition, including abrupt c‐axis contraction, stress accumulation, and increased interfacial/diffusion‐related polarization [65, 71]. Cross‐sectional SEM images of the electrodes and secondary particles before and after cycling provide clear evidence of this instability (Figure S4a–h). Prior to cycling, both the LNO and LNO‐HE2 electrodes exhibited relatively intact spherical secondary particles distributed throughout the electrode cross‐sections (Figure S4a, c). However, after 100 charge–discharge cycles, the pristine LNO electrode exhibited more pronounced collapse and fragmentation of the secondary particles, indicating severe deterioration of the particle structure within the electrode cross‐section (Figure S4b, f). Specifically, the cycled LNO secondary particle (Figure S4f) exhibited a partially collapsed morphology accompanied by increased internal porosity, namely enlarged voids between primary particles, leading to reduced particle continuity. This suggests that repeated Li^+^ extraction/insertion induces significant chemomechanical degradation in the undoped LNO electrode. Conversely, although the LNO‐HE2 electrode exhibited slight morphological degradation after 100 cycles, the secondary particles maintained a denser and more continuous structure relative to pristine LNO (Figure S4g, h). This improved particle structural stability suggests that HE doping alleviates local stress concentrations during cycling and suppresses the collapse of secondary particles. The superior morphological stability of LNO‐HE2 can be attributed to its denser and more uniform secondary‐particle structure, which may reduce electrolyte penetration into the particle interior and mitigate crack propagation during repeated Li^+^ extraction/insertion [72].

To further corroborate the in situ XRD observations, the ex situ XRD patterns of LNO‐HE2 were collected at six representative charge–discharge states (Steps 1–6; Figure S4i). As shown in Figure S4j, all patterns are indexed to the layered rhombohedral *R‐3m* structure, without the emergence of discernible secondary reflections attributable to the spinel‐ or rock‐salt‐type phases, indicating that the framework is largely preserved across the sampled states [73]. Consistent with the in situ (003) evolution, the magnified (003) reflection (Figure S4k) exhibits the state‐of‐charge‐dependent peak shifts, reflecting reversible modulation of the c‐axis lattice parameter (i.e., lattice breathing) during Li extraction/insertion. In parallel, the position and line shape of the (104) reflection (Figure S4l) changes concomitantly, suggesting anisotropic lattice responses involving both interlayer spacing and in‐plane distortion. Notably, the peak shifts remain largely reversible upon discharge, although the slight broadening of the peak and incomplete recovery of the peak shape imply residual strain and accumulated disorder, in line with the non‐fully reversible lattice response observed near the high‐voltage H2–H3 transition. Overall, the ex situ snapshots complement the operando results by confirming that the primary structural evolution of LNO‐HE2 is governed by reversible lattice breathing rather than irreversible phase transformation, supporting the enhanced structural robustness under high‐voltage operation [24, 71, 74].

**FIGURE 7:**
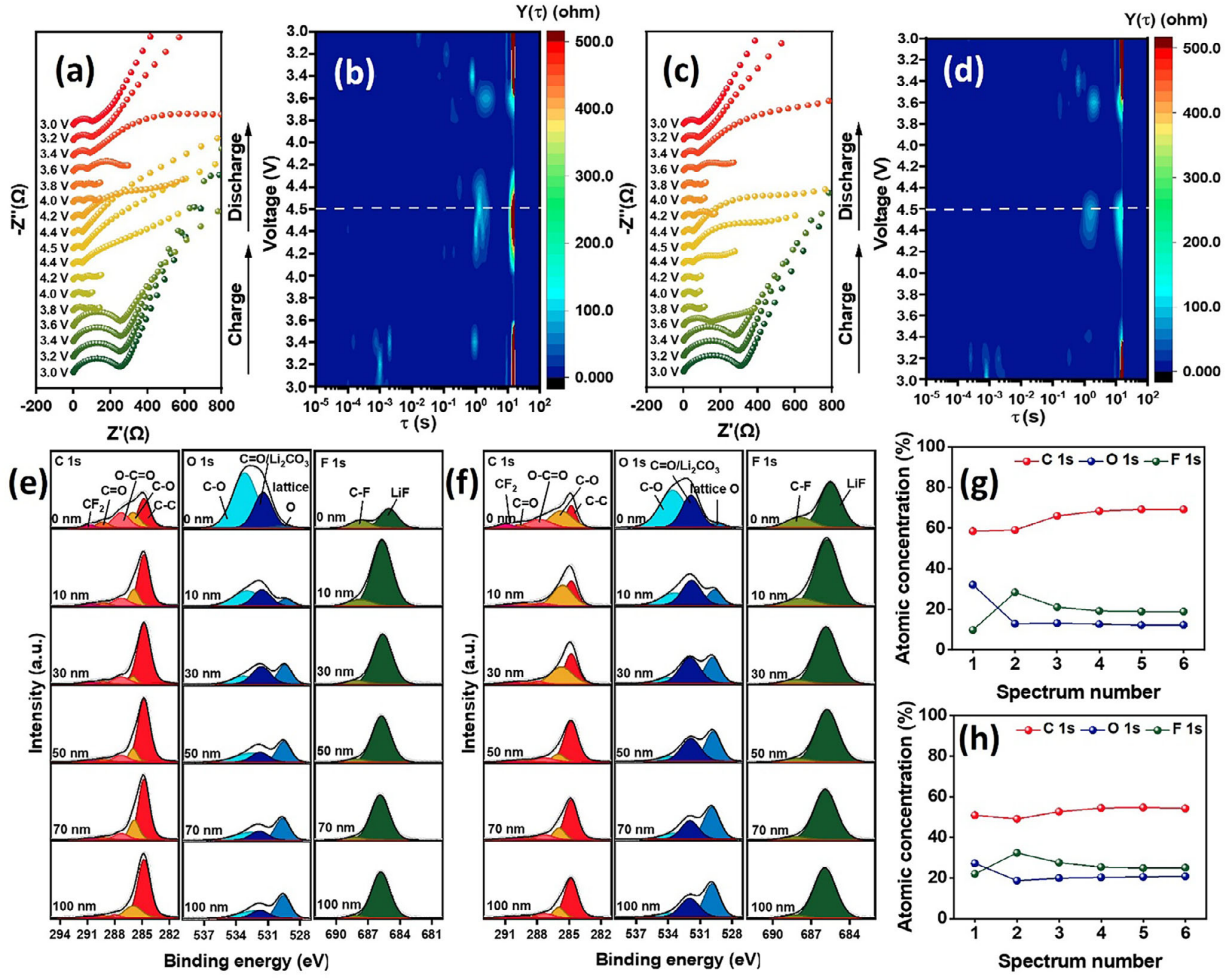
In situ EIS/DRT and XPS depth profiling analyses of LNO and LNO‐HE electrodes. In situ EIS results of (a, b) LNO and (c, d) LNO‐HE2 measured at selected voltages during charge and discharge. (a, c) Nyquist plots, and (b, d) DRT spectra. XPS depth‐profiles of the CEI formed on the LNO and LNO‐HE2 electrodes after 100 cycles: high‐resolution C 1s, O 1s, and F 1s spectra of (e) LNO and (f) LNO‐HE2 collected at sputtering depths of 0, 10, 30, 50, 70, and 100 nm. Atomic concentration profiles of C 1s, O 1s, and F 1s for (g) LNO and (h) LNO‐HE2 electrodes.

To compare the voltage dependence of the electrode–electrolyte interfacial reactions, in situ electrochemical impedance spectroscopy (EIS) was conducted for the LNO and LNO‐HE2 electrodes at selected states of charge between 3.0 and 4.5 V during both charge and discharge. The corresponding distribution of relaxation time (DRT) spectra were also collected (Figure 7). In the Nyquist plots (Figure 7a, c), the mid‐frequency semicircular arc becomes more pronounced and increases in diameter with increasing voltage [75]. In the ranges of 3.8–4.2 V during charge and 4.2–3.6 V during discharge, two arcs are partially resolved, whereas at 4.4–4.5 V, the arcs strongly overlap, appearing as a single, broadened semicircle. This morphological evolution is mirrored in the DRT maps (Figure 7b, d): at intermediate voltages, the impedance response is distributed across distinct relaxation‐time (τ) regions, while the response becomes more concentrated and less separable at high voltages [76].

Compared with LNO, LNO‐HE2 shows a relatively attenuated increase in the high‐voltage impedance response, as reflected by the mid‐frequency arc and DRT intensity/extent. This trend is consistent with the mitigated structural evolution observed by in situ XRD in the high‐voltage H2–H3 transition regime, where LNO‐HE2 exhibits smaller (003) peak shift/distortion (and splitting) and smaller c‐axis contraction amplitude (*Δc*) and residual (non recovered) components than LNO. Additionally, LNO‐HE2 tends to exhibit a higher apparent D_Li_^+^ value, estimated from the low‐frequency Warburg diffusion component, than LNO (Table S3), suggesting that diffusion‐limited polarization may be comparatively alleviated across the charge–discharge voltage window [77]. Collectively, these results indicate that multicomponent (HE) doping correlates with moderated impedance build‐up under high‐voltage operation, in conjunction with alleviated structural instability.

To further elucidate the chemical nature of the cathode–electrolyte interphase (CEI) formed on the cathode surface, XPS depth profiling was performed on the LNO and LNO‐HE2 electrodes after 100 cycles, as shown in Figure 7e–h. In both samples, C‐, O‐, and F‐containing species were detected at the outermost surface, indicating the formation of a CEI layer through oxidative electrolyte decomposition during repeated charge–discharge cycling [78]. However, the depth‐dependent compositional evolution differed distinctly between the two electrodes. For the LNO electrode (Figure 7e), the C 1s signal remained pronounced even after sputtering [79], and the atomic concentration profile (Figure 7g) showed that carbon was the dominant component, accounting for approximately 60–70 at%. This result suggests that a substantial amount of organic carbonate‐derived decomposition products or carbonaceous species accumulated on the surface and near‐surface region of the cycled LNO electrode. Such a carbon‐rich CEI can be associated with continuous electrolyte oxidation and may impede Li^+^ transport, thereby increasing interfacial resistance and polarization [80]. In addition, the O 1s signal was initially strong at the surface but decreased sharply upon sputtering [81], reflecting the preferential removal of oxygen‐containing decomposition products from the LNO electrode surface. In contrast, the LNO‐HE2 electrode (Figure 7f, h) exhibited a relatively lower carbon content than LNO, while F, and O species were detected in a more balanced manner. In particular, the F 1s signal remained relatively stable even after sputtering [82], suggesting that F‐rich inorganic CEI components, such as LiF‐like species, were distributed within the near‐surface region [83]. This behavior indicates that HE doping may suppress excessive organic electrolyte decomposition during cycling and promote the formation of a relatively stable inorganic‐rich CEI [84]. Therefore, the XPS depth profiling results suggest that HE doping mitigates the excessive accumulation of carbonaceous CEI species on the LNO cathode surface and facilitates the formation of a more stable F/Li‐containing inorganic CEI. This finding is consistent with the attenuated impedance growth and enhanced interfacial stability of the LNO‐HE2 electrode observed by in situ EIS. Consequently, the more stable CEI structure induced by HE doping is considered to contribute to improved cycling stability by suppressing electrolyte decomposition and interfacial resistance growth under high‐voltage operation.

**FIGURE 8:**
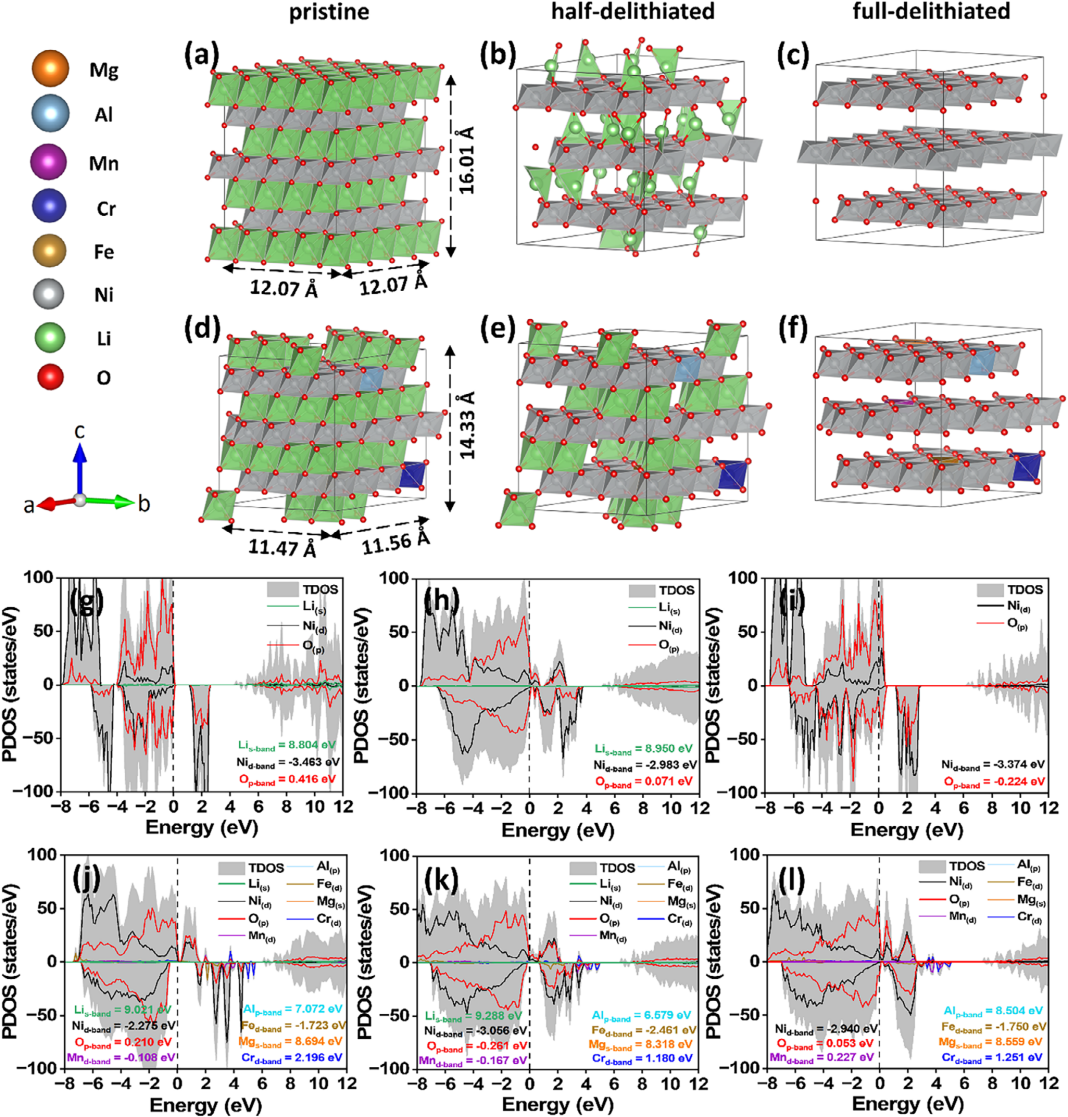
Bulk structures of (a–c) LNO and (d–f) LNO‐HE2, projected density of states (PDOS) of (g–i) LNO and (j–l) LNO‐HE2.

To gain atomistic insight into the structural stability and the role of multi‐element substitution in Ni‐rich layered oxides, DFT calculations were performed. This enables direct comparison of the lattice parameters, phase stability, and local electronic environments between pristine LNO and HE‐doped analogues, thereby providing a theoretical foundation to complement the experimental observations. The bulk crystal structure of LNO consists of 48 Li, 48 Ni, and 96 O atoms, with the following lattice parameters: *a* = b = 12.07 Å, *c* = 16.01 Å, *α* = *β* = 90°, and *γ* = 120° (Figure 8a–c). The simulated XRD pattern agrees well with the experimental result (Figure S5), confirming the validity of the structural model. To construct the HE models, multiple transition metals (Mn, Al, Fe, Mg, and Cr) were randomly substituted at the Ni sites (Figure S6). Among the 10 designed structures, the configuration of LNO‐HE2 is identified as the most stable, containing 48 Li, 43 Ni, and 96 O atoms as well as one atom each of Mn, Al, Fe, Mg, and Cr. The optimized lattice parameters are *a* = 11.56 Å, *b* = 11.47 Å, and *c* = 14.33 Å (*α* = *β* = 90° and *γ* = 120°) (Figure 8d–f).

To further elucidate the electronic origin of enhanced stability, projected density of states (PDOS) calculations were conducted (Figure 8g–l). For the pristine LNO, partial delithiation led to an upshift of the Ni *d*‐band center from −3.463 to −2.983 eV, indicating stronger Ni─O hybridization and an increased Ni oxidation state (Ni^3+^→ Ni^4+^). Upon full delithiation, the Ni *d*‐band center shifts downward to –3.374 eV due to electronic saturation of the Ni *d*‐orbitals and oxygen participation in redox activity. Correspondingly, the O *p*‐band shifts from 0.416 to −0.224 eV below the Fermi level upon delithiation. Such a shift indicates that oxygen redox activation is significant when Ni reaches higher oxidation states. Although oxygen participation is beneficial for capacity, it can lead to structural instability due to oxygen loss.

In contrast, for LNO‐HE2, the Ni *d*‐band center shifts downward from −2.275 to −3.056 eV upon partial delithiation, reflecting stabilization of the Ni electronic states via redox participation of the neighboring multivalent cations, likely Fe^3+^→ Fe^4+^ and Mn^3+^→ Mn^4+^. Upon full delithiation, the Ni *d*‐band center slightly shifts upward to −2.940 eV due to a more stable and balanced electronic configuration relative to the pristine system. Notably, the Al and Mg electronic states are situated far from the Fermi level and do not participate in the redox process; instead, they contribute to structural stabilization. Similarly, Cr exhibits limited redox activity and primarily contributes to entropy‐driven stabilization.

Notably, the O *p*‐band in the doped LNO‐HE2 initially shifts from 0.210 to −0.261 eV upon partial delithiation, indicating early oxygen participation. However, unlike the behavior observed in pristine LNO, the O *p*‐band shifts back toward the Fermi level to 0.053 eV upon full delithiation. This suggests that oxygen redox is moderated by the presence of multivalent cations, which distributes the charge compensation and prevents excessive oxidation of oxygen. Thus, the entropy‐stabilized multicomponent lattice enables a balanced redox mechanism by reducing the oxygen participation that can enhance structural stability during full delithiation.

To quantitatively correlate the role of individual dopants, the evolution of the Ni *d*‐band and O *p*‐band centers during delithiation were compared. In the pristine LNO, the Ni *d*‐band center initially shifted upward by approximately 0.48 eV, indicating strong oxidation of Ni. Conversely, the doped system exhibited a downward shift of approximately 0.78 eV, reflecting the stabilization of the Ni states due to the involvement of multivalent dopants.

Among the dopants, Mn and Fe play a dominant role in redox kinetics through their variable oxidation states, which reduces the oxidation burden on Ni. This is evidenced by the reduced magnitude of the Ni *d*‐band shift in the doped system compared to that in the pristine counterpart. In contrast, Al and Mg exhibit electronic states far from the Fermi level, confirming their non‐redox nature and their role in structural stabilization by suppressing electronic fluctuations. Chromium exhibits limited redox activity and contributes to the entropy stabilization of the lattice. This quantitative comparison confirms that Mn and Fe enhance redox flexibility, while Al, Mg, and Cr contribute to structural and thermodynamic stabilization.

**FIGURE 9:**
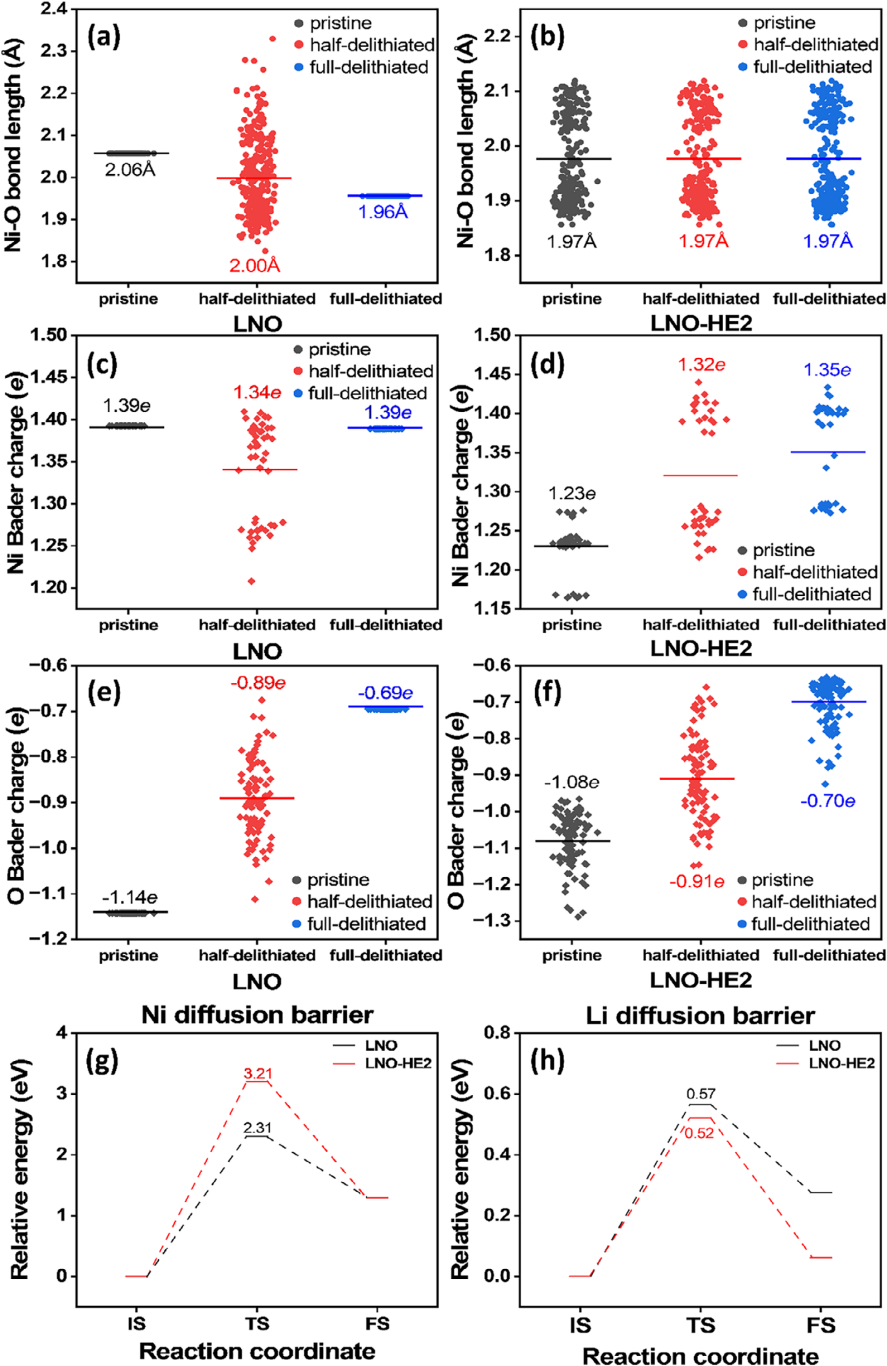
(a, b) Ni─O bond length, (c, d) Ni net Bader charge, (e, f) O net Bader charge at pristine, half‐delithiated, and fully‐delithiated states (blue lobes represent charge accumulation and red lobes indicate charge depletion). (g, h) Ni and Li diffusion barriers for LNO and LNO‐HE2, evaluated at the initial state (IS), transition state (TS), and final state (FS).

To examine the role of HE effects, the distributions of Ni─O bond lengths in the LNO and LNO‐HE2 structures are compared in Figure 9a, b. For the LNO structure, the Ni─O bond length distribution varies from 2.06 to 1.96 Å upon delithiation. Such a broad distribution indicates enhanced lattice relaxation during Li extraction, which may facilitate Ni migration into Li layers. By contrast, the LNO‐HE2 structure shows an average Ni─O bond length of 1.97 Å upon delithiation, which is shorter and more uniform compared with that of LNO. This reduced fluctuation indicates enhanced Ni–O bond rigidity, thereby suppressing the lattice relaxation associated with Ni migration and oxygen release. The Bader charge was calculated to demonstrate the influence of HE multivalent cation doping on the LNO and LNO‐HE2 structures, as shown in Figure 9c–f. For the LNO structure, the average charge of Ni atoms is 1.39e, which decreases to 1.34e during half‐delithiation, indicating an initial Ni reduction. Upon full delithiation, the Ni charge increases again to 1.39e, reflecting a re‐oxidation process. By contrast, the LNO‐HE2 structure shows an increasing trend in Ni charge from 1.23e to 1.35e, indicating progressive oxidation upon Li extraction. The lower initial Ni charge suggests electron donation from multivalent cations. Both LNO and LNO‐HE2 exhibit an increase in the Bader charge on oxygen during delithiation, confirming that lattice oxygen contributes to the oxidation process. However, the oxygen in LNO‐HE2 remains more negatively charged under both partial and full delithiation, suggesting stabilization of the O‐ligand framework by multidopants.

Furthermore, charge density difference analysis was conducted for LNO and LNO‐HE2 to examine the charge redistribution and redox behavior, as shown in Figure S7a–d. For LNO, the isosurface plot reveals that charge redistribution is localized primarily around the Ni─O framework upon partial Li extraction. Upon full delithiation, charge depletion near the Ni site is less pronounced compared with that near the O site, indicating that strong oxygen oxidation dominates at this stage, which ultimately leads to structural degradation. By contrast, LNO‐HE2 shows a more balanced redox process with charge compensation distributed more evenly across multiple cations during both partial and full delithiation. Moreover, the reduced oxygen‐localized charge depletion contributes to the enhanced structural stability.

To gain further insight into the structural stability and ion transport behavior, nudged elastic band (NEB) calculations were performed to evaluate the migration barriers of Ni and Li in LNO and LNO‐HE2, as shown in Figures 9g, h and S7e, f. LNO exhibits a diffusion barrier of 2.31 eV, while HE‐doped LNO‐HE2 shows a significantly higher barrier of 3.21 eV. This indicates that the incorporation of multiple cations effectively suppresses Ni migration into the Li layers, thereby mitigating Ni/Li cation mixing during cycling and enhancing structural integrity.

In contrast, Li diffusion exhibits an opposite trend. The diffusion barrier of Li in the LNO‐HE2 structure (0.52 eV) is slightly lower than that in LNO (0.57 eV), suggesting that Li^+^ migration is facilitated upon HE doping. This behavior can be rationalized by the stabilization of the Ni valence state via multivalent cation substitution, which weakens the repulsive interaction arising from lowering the energy barrier and enabling faster diffusion. Overall, HE doping provides a dual benefit by suppressing detrimental Ni migration while simultaneously enhancing Li^+^ mobility, thereby improving the structural stability and electrochemical kinetics.

**FIGURE 10:**
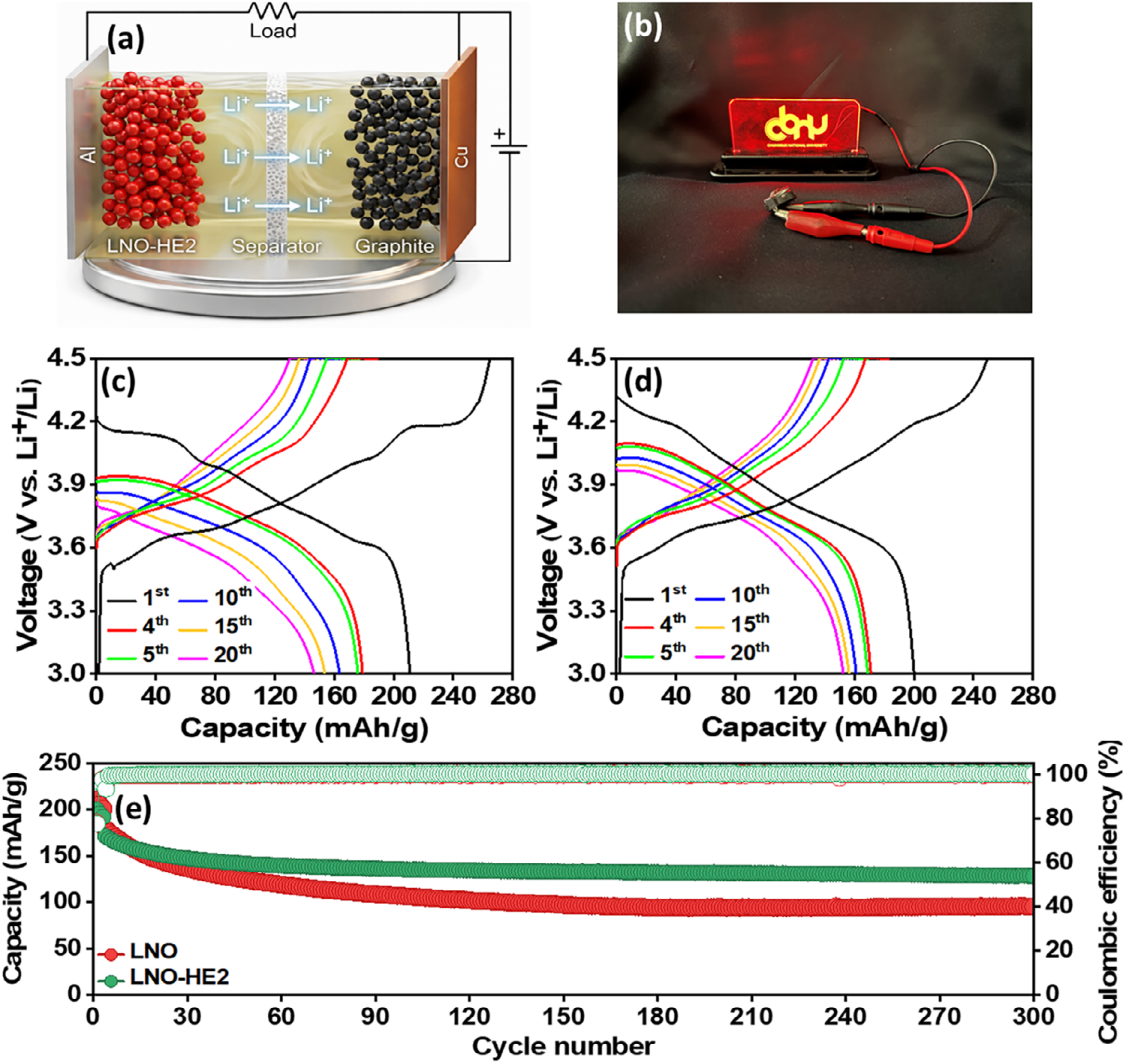
Electrochemical performance of coin‐type full cells using graphite as the anode material and LNO or LNO‐HE2 as the cathode material. (a) Schematic illustration of the full‐cell configuration. (b) Photograph demonstrating an LED powered by the assembled full cell. Galvanostatic charge–discharge (GCD) profiles at selected cycle numbers for full cells with (c) LNO and (d) LNO‐HE2 as the cathode. (e) Cycling performance of the full cell measured at 0.5C.

To evaluate the cycle stability and polarization behavior under practical operating conditions, coin‐type graphite||cathode full cells (N/p = 1.25) were assembled using LNO or LNO‐HE2 as the cathode (Figure 10). Figure 10a illustrates the full‐cell configuration, and Figure 10b demonstrates an LED powered by the assembled full cell, in which LNO‐HE2 is used as the electrode. Figure 10c, d compare the voltage–capacity profiles of the graphite||LNO and graphite||LNO‐HE2 full cells at selected cycles. The charge/discharge curves for the graphite||LNO full cell exhibit a progressive decrease in discharge capacity and a noticeable increase in voltage hysteresis with cycling (Figure 10c). The enlarged gap between the charge and discharge profiles indicates the accumulation of polarization and increased kinetic resistance during repeated cycling. In contrast, the graphite||LNO‐HE2 full cell maintains more stable GCD profiles with a smaller voltage hysteresis increase and better retention of the discharge curve shape (Figure 10d). This suggests that LNO‐HE2 effectively suppresses polarization accumulation and maintains improved electrochemical reversibility under full‐cell operating conditions. These GCD results are consistent with the half‐cell behavior shown in Figure 5, further supporting that HE doping mitigates electrode/electrolyte interfacial degradation and structural deterioration during cycling [63].

As shown in Figure 10e, both cells were pre‐cycled at 0.1C and subsequently cycled at 0.5C for long‐term testing. The initial discharge capacities at 0.1C are 210.99 and 200.13 mAh g^−1^ for the graphite||LNO and graphite||LNO‐HE2 full cells, respectively. During prolonged cycling at 0.5C, the capacity retention after 100 cycles was 59% for LNO and 79% for LNO‐HE2, demonstrating the superior durability of the LNO‐HE2 cathode. In addition, both cells maintain high coulombic efficiencies throughout cycling. The cycling performance at 0.3C and GCD values at 0.1C–0.3C are provided in Supporting Information (Figure S8). Overall, LNO‐HE2 exhibits improved electrochemical stability in graphite‐based full cells by mitigating polarization accumulation, while maintaining higher coulombic efficiency and enhanced long‐term durability compared with LNO.

## 3 Conclusions

This study demonstrates that HE doping utilizing Mn, Al, Fe, Mg, and Cr via a simple and scalable 3D mixing process is an effective strategy for stabilizing Co‐free Ni‐rich LiNiO_2_ cathode materials. The optimized LNO‐HE2 composition exhibited improved layered ordering, reduced Li/Ni intermixing, and enhanced morphological uniformity, as compared with pristine LNO. The high‐entropy dopants effectively mitigated polarization growth, improved Li^+^ transport kinetics, and suppressed structural degradation during repeated Li^+^ extraction/insertion. Therefore, LNO‐HE2 maintained a stable layered framework and secondary‐particle structure, whereas pristine LNO exhibited severe particle collapse, internal void formation, and loss of particle continuity. Consequently, LNO‐HE2 achieved a capacity retention of 79% after 100 cycles, markedly higher than the 51% retained by pristine LNO. Furthermore, the graphite||LNO‐HE2 full cell exhibited improved long‐term durability, confirming the practical applicability of the proposed strategy under full‐cell conditions. These results demonstrate that 3D‐mixer‐assisted HE doping is a practical and scalable approach for simultaneously improving the structural stability, morphological robustness, and electrochemical durability of Co‐free Ni‐rich layered cathodes.

## 4 Experimental Section

Detailed information on materials used, material synthesis, electrode fabrication, cell assembly, material characterization, electrochemical measurements, and computational method is provided in the Supporting Information.

## Author Contributions

**Seung Ri Kim**: Investigation, Methodology, Visualization, Writing – Original Draft. **Eun Mi Kim**: Formal analysis, Writing – Original Draft, Writing – Review & Editing, Resources. **Thillai Govindaraja Senthamaraikannan**: DFT calculation and related content preparation. **Jun Jae Myeong**: Validation, Data Curation. **Hyung Do Kim**: Visualization, Validation. **Dong‐Hee Lim**: DFT calculations and related content review & editing. **Sang Mun Jeong**: Conceptualization, Supervision, Funding acquisition, Writing – review & editing, Project Administration.

## Conflicts of Interest

The authors declare no conflict of interest.

## Supporting information




**Supporting File**: advs76214‐sup‐0001‐SuppMat.docx.

## Data Availability

The data that support the findings of this study are available from the corresponding author upon reasonable request.
