# Supplementary material for: 3D‐Mixer‐Assisted High‐Entropy Doping of LiNiO2 for Co‐Free Ni‐Rich Cathodes in Lithium‐Ion Batteries
Source: Adv Sci (Weinh). 2026 Jun 22:e76214. Online ahead of print. doi: 10.1002/advs.76214 (PMC13336411; doi:10.1002/advs.76214)
Supplement: Supplementary file 1 — Supporting File: advs76214‐sup‐0001‐SuppMat.docx. [file ADVS-9999-e76214-s001.docx]

Copyright WILEY-VCH Verlag GmbH & Co. KGaA, 69469 Weinheim, Germany, 2018.

Supporting Information

3D-Mixer-Assisted High-Entropy Doping of LiNiO_2_ for Co-Free Ni-Rich Cathodes in Lithium-Ion Batteries

Seung Ri Kim^†^, Thillai Govindaraja Senthamaraikannan^†^, Jun Jae Myeong, Hyung Do Kim, Dong-Hee Lim, Eun Mi Kim*, and Sang Mun Jeong*

**Experimental Section**

**Materials**

All chemicals were used as received without further purification. Lithium hydroxide monohydrate (LiOH⋅H_2_O, 99.995%, Sigma-Aldrich), Nickel(II) nitrate hexahydrate (Ni(NO_3_)_2_⋅6H_2_O, ≥97 %, Sigma-Aldrich), Manganese(II) nitrate tetrahydrate (Mn(NO_3_)_2_·4H_2_O, ≥97 %, Sigma-Aldrich), Aluminum nitrate nonahydrate (Al(NO_3_)_3_⋅9H_2_O, ≥98%, Sigma-Aldrich), Iron(III) nitrate nonahydrate (Fe(NO_3_)_3_⋅9H_2_O, 98 %–101%, Alfa Aesar), Magnesium nitrate hexahydrate (Mg(NO_3_)_2_⋅6H_2_O, 99 %, Sigma-Aldrich), Chromium(III) nitrate nonahydrate (Cr(NO_3_)_3_⋅9H_2_O, 99%, Sigma-Aldrich), and anhydrous ethyl alcohol (C_2_H_6_O, 99.9%, SAMCHUN) were used in this study.

**Synthesis of Materials**

Ni(NO_3_)_2_⋅6H_2_O (7 g) was dissolved in ethanol (70 mL) and transferred to a 100 mL autoclave for hydrothermal treatment. The reaction was performed at 180 ℃ for 24 h, and the product was collected by filtration, thoroughly washed, and vacuum-dried at 80 °C for 12 h to obtain α-3Ni(OH)_2_·2H_2_O powder.

A HE precursor solution was prepared by dissolving Mn(NO_3_)_2_·4H_2_O, Al(NO_3_)_3_⋅9H_2_O, Fe(NO_3_)_3_⋅9H_2_O, Mg(NO_3_)_2_⋅6H_2_O, and Cr(NO_3_)_3_⋅9H_2_O in ethanol at an equimolar ratio (1:1:1:1:1). The prepared HE solution was homogenized with the previously obtained α-3Ni(OH)_2_·2H_2_O at a Ni:HE molar ratio of 1:*x* (*x* = 0.05, 0.10, 0.15) using a 3D mixer at 72 rpm for 2 h, and then dried in an oven at 60 °C. The dried doped mixture was homogenized with 1.05 mol of LiOH·H_2_O and 10 mL of ethanol using a 3D mixer (72 rpm) for 2 h, and then dried at 60 °C. The mixture was subsequently calcined under an oxygen atmosphere at a heating rate of 2 °C min^-1^: first at 530 °C for 3 h, followed by 730 °C for 12 h. The calcined powder (1 g) was dispersed in 10 mL of deionized water and ultrasonicated for washing. To compensate for lithium loss during washing, LiOH·H_2_O (5 wt.%) was added before the second calcination. The second calcination was performed at 700 °C for 6 h (heating rate: 2 °C min^-1^), yielding the final HE-doped cathode material. For comparison, LNO was synthesized, and the synthesis procedure was identical to that of the HE-doped sample except that no HE elements were introduced. The synthesis procedure is illustrated in **Figure S9**.

**Battery Assembly**

A cathode slurry was prepared using the synthesized active material, LNO-HE, as 80 wt.%, conductive carbon black as 10 wt.%, and poly(vinylidene fluoride) (PVDF) binder as 10 wt.%. PVDF was dissolved in N-methyl-2-pyrrolidone (NMP) prior to mixing. The resulting slurry was cast onto aluminum foil current collectors via the doctor-blade method and dried at 60 °C for 2 h. The dried electrodes were calendered to an 80% compaction ratio to optimize pore structure and electrode density, and subsequently punched into 13 mm-diameter discs. To remove residual solvent, the punched electrodes were vacuum-dried at 110 °C for 12 h.

The fabricated electrodes were assembled into CR2032-type half-cells for electrochemical evaluation. CR2032 half-cells were assembled using lithium metal (16 mm in diameter) as the counter/reference electrode, a polypropylene separator (Celgard 2400), and an electrolyte of 1.2 M LiPF_6_ in EC:EMC (3:7, v/v) with 2 wt.% vinylene carbonate. All assembly processes were performed in a high-purity Ar-filled glovebox, with O_2_ and H_2_O levels maintained below 0.01 ppm.

**Material Characterization and Electrochemical Measurements**

The crystal structure of the synthesized LNO-HE cathodes was analyzed via powder XRD using a Rigaku Ultima IV diffractometer (Rigaku, Japan) with Cu Kα radiation (λ = 1.54 Å). Diffraction patterns were collected over 2θ range of 10°–80° at a scan rate of 1° min^-1^. Morphological and microstructural analyses were conducted employing field-emission scanning electron microscopy (FE-SEM, LEO-1530, Carl Zeiss, Germany), energy dispersive X-ray spectroscopy (EDS, Carl Zeiss, Germany), and HR-TEM (Libra 200MC, 200 kV, Carl Zeiss, Germany). FE-SEM and EDS were used to evaluate particle size, shape, and distribution, whereas HR-TEM was employed to comprehensively investigate lattice fringes, grain boundaries, and microdefects. Elemental composition and surface chemical states were characterized via XPS (PHI Quantera-II, Ulvac-PHI, Chigasaki, Japan). Additionally, cross-sectional SEM and ex situ XRD analyses were conducted on LNO and LNO-HE cathodes. For cross-sectional imaging, cells were cycled 100 times at 0.3C within 3.0–4.5 V, and electrode cross sections were observed using FE-SEM (LEO-1530, Carl Zeiss, Germany). For ex situ XRD, electrodes charged/discharged at 0.1C within 3.0–4.5 V were analyzed using a Rigaku Ultima IV diffractometer (Rigaku, Japan) with Cu Kα radiation (λ = 1.54 Å) over 2θ range of 10°–80° at a scan rate of 1° min^-1^. In-situ XRD measurements were performed using a PANalytical Empyrean diffractometer (Malvern PANalytical), and the diffraction patterns were recorded in the 2θ range of 15°–25° at a scan rate of 1° min^-1^.

In-situ electrochemical impedance spectroscopy (EIS) measurements were carried out over a frequency range of 0.01–100 kHz with an AC amplitude of 10 mV using a WBCS-3000L system (WonATech, Korea) coupled with a ZIVE SP1 analyzer. The electrochemical performance of the coin cells was evaluated using a WBCS-3000L galvanostat/potentiostat (WonATech, Korea) through CV and GCD analyses. CV was performed between 2.6–4.6 V at a scan rate of 0.05 mV s^-1^. GCD cycling was conducted within 3.0–4.5 V (vs. Li^+^/Li) at a current rate of 0.3C for 300 cycles. Rate capability was assessed at current rates ranging from 0.1C to 5C. GITT measurements were performed after a single formation cycle at 0.1C within 3.0-4.5 V. The coin full cells were assembled using the same components from the same manufacturer as those employed for the half cells. Commercial graphite was used as the anode material, and the N/P ratio was controlled to be 1.25. Galvanostatic charge–discharge (GCD) cycling was performed in a voltage range of 3.0–4.5 V at 0.5C. XPS depth profiling of the cycled electrodes was performed using an X-ray photoelectron spectrometer (PHI GENESIS, Ulvac-PHI) equipped with a monochromatic Al Kα X-ray source. The X-ray beam size was 100 μm, and the spectra were collected at a take-off angle of 45° with charge neutralization. Survey spectra were acquired with a pass energy of 280 eV and a step size of 1.0 eV, while high-resolution narrow spectra were collected with a step size of 0.05 eV. Depth profiling was conducted by Ar ion sputtering at 3 kV with a raster size of 2 × 2 mm². The sputtering rate was calibrated to 1.5 Å s⁻¹ using SiO₂ as a reference, and XPS spectra were collected at SiO₂-equivalent depths of 0, 10, 30, 50, 70, and 100 nm.

**Computational Methods**

DFT calculations were performed using the Vienna Ab-initio Simulation Package (VASP)^[1-4]^with the projected-augmented wave (PAW) method.^[5-6]^ The exchange-correlation functional was treated within the generalized gradient approximation (GGA) using the Perdew–Burke–Ernzerhof (PBE) parameterization to accurately describe the core–valence interactions. A plane-wave basis set with a cutoff energy of 520 eV was applied for all calculations.^[7]^ In the GGA + U approach proposed by Dudarev et el., the effective U values were set to 3.8 eV for Cr, 4.3 eV for Fe, 4.0 eV for Mn, and 6.2 eV for Ni.^[8]^ Brillouin zone integration was performed using a 2 $\times$ 2 $\times$ 1 k-point mesh for LNO and LNO-HE2 bulk calculations.^[9]^ Furthermore, the energy barriers for Li and Ni migration were determined using the climbing nudged elastic band (Cl-NEB) method.


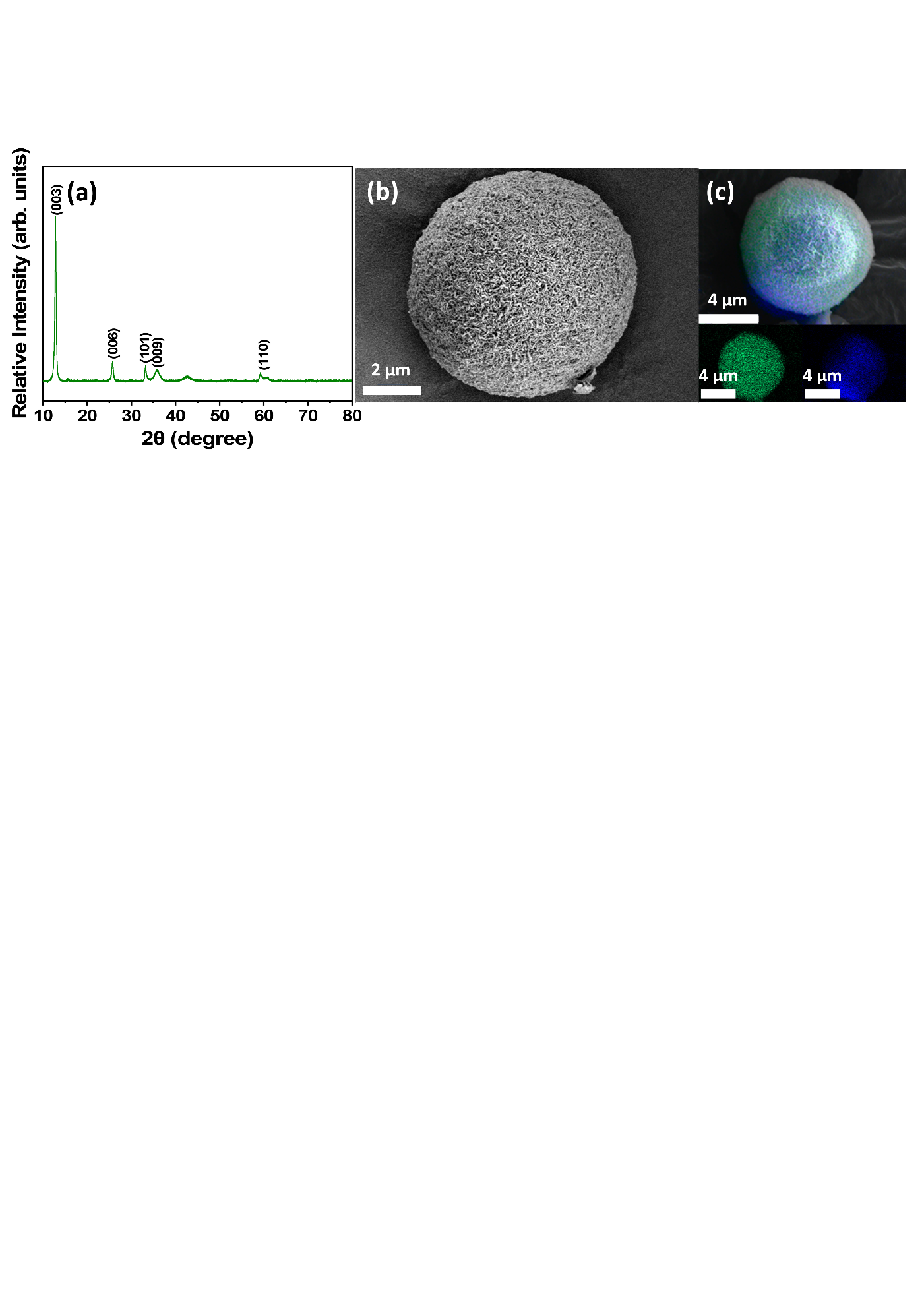

**Figure S1**. Characterization of the α‑3Ni(OH)_2_·2H_2_O precursor. (a) XRD pattern confirming the typical layered α-phase structure, (b) SEM image showing the spherical morphology of the secondary particle, (c) EDS mapping images.

**
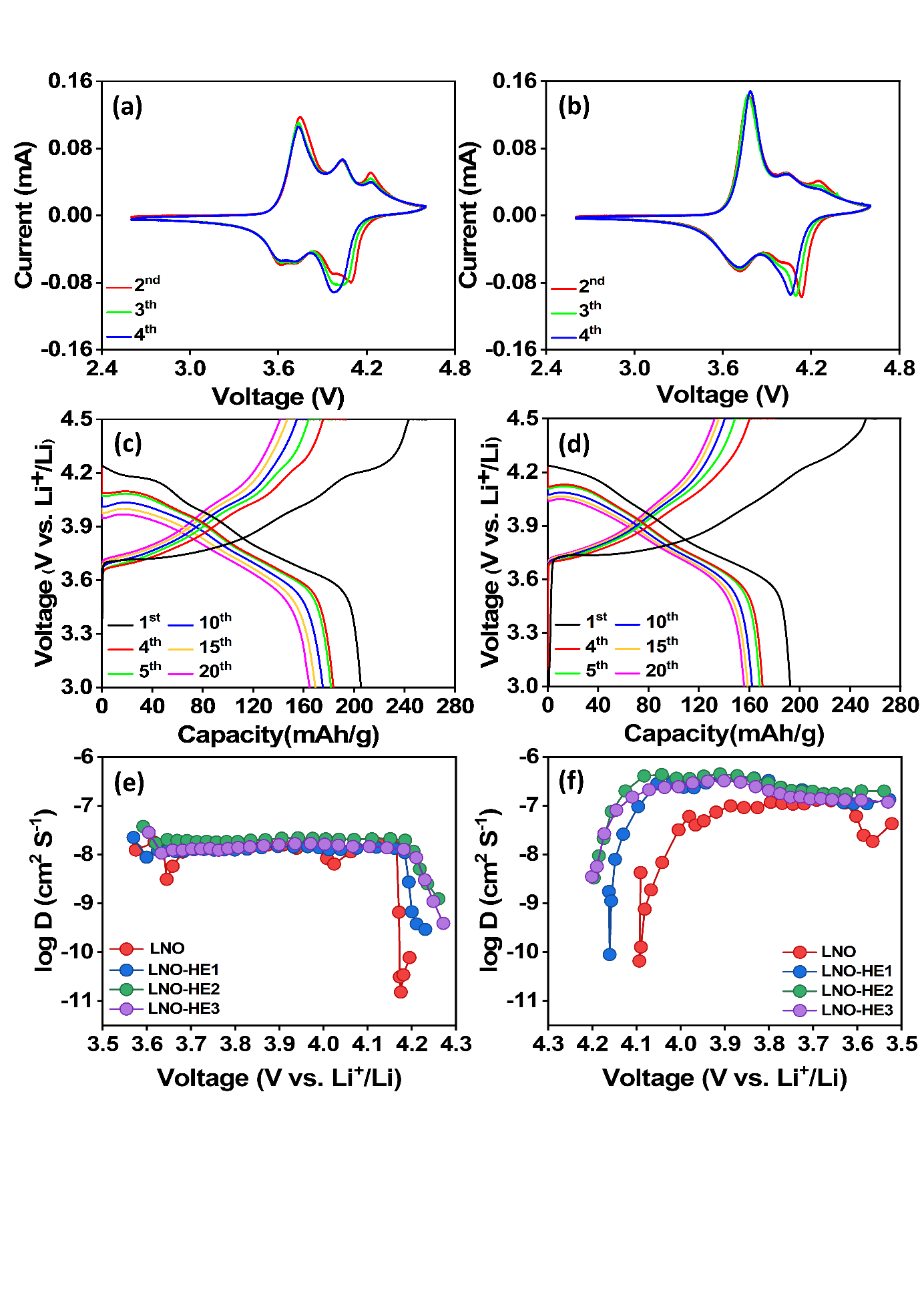
**

**Figure S2**. CV and GCD curves of (a, c) LNO-HE1 and (b, d) LNO-HE3. The measurements were conducted in the voltage range of 2.6–4.6 V (vs. Li^+^/Li) at a scan rate of 0.05 mV s^-1^. (e, f) Lithium-ion diffusion coefficients obtained from galvanostatic intermittent titration technique (GITT) measurements during (e) charging and (f) discharging at 0.1C. Pulse duration was 20 min and the relaxation time was 40 min.


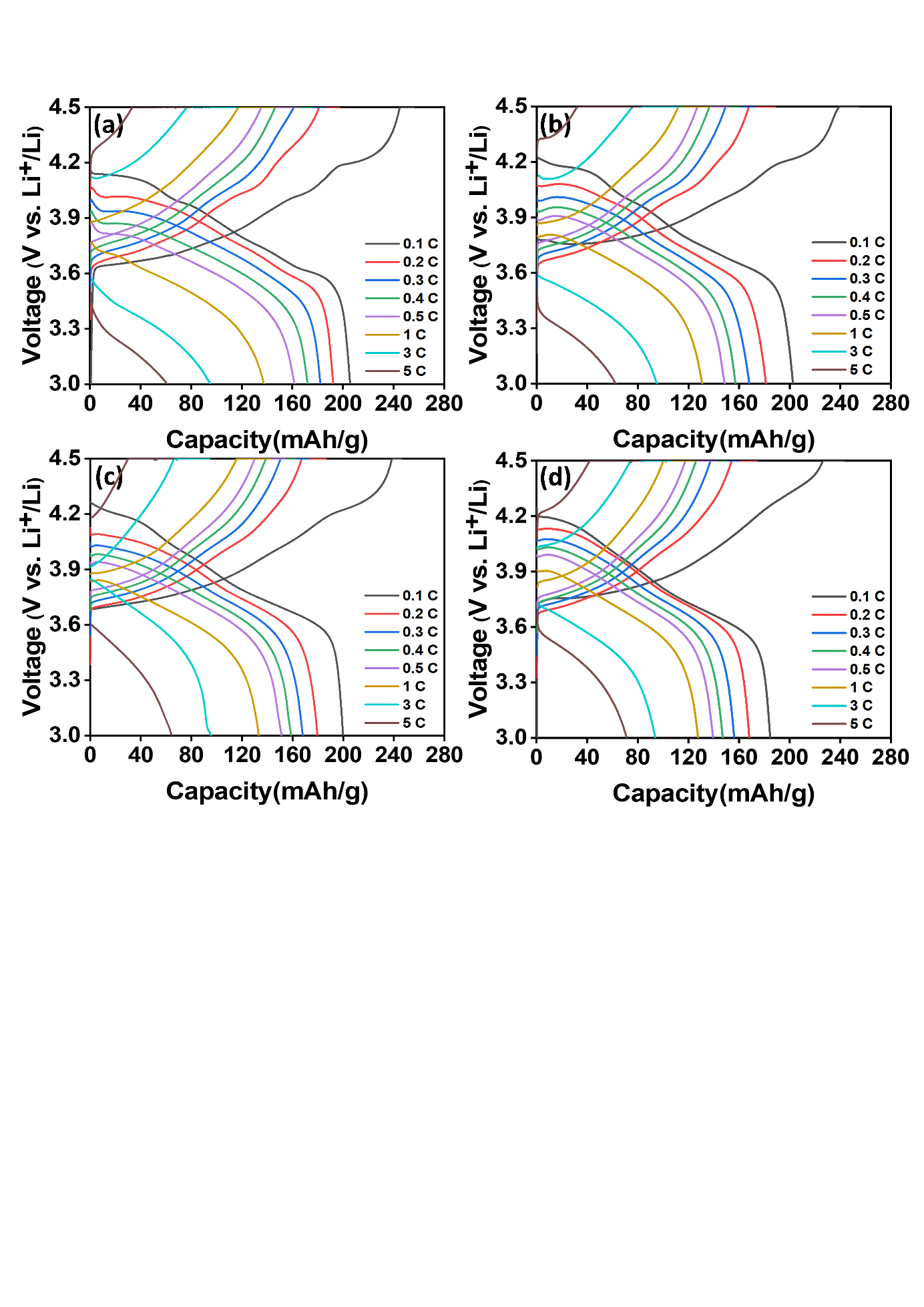


**Figure S3.** Rate-dependent GCD curves of (a) LNO, (b) LNO-HE1, (c) LNO-HE2, and (d) LNO-HE3.


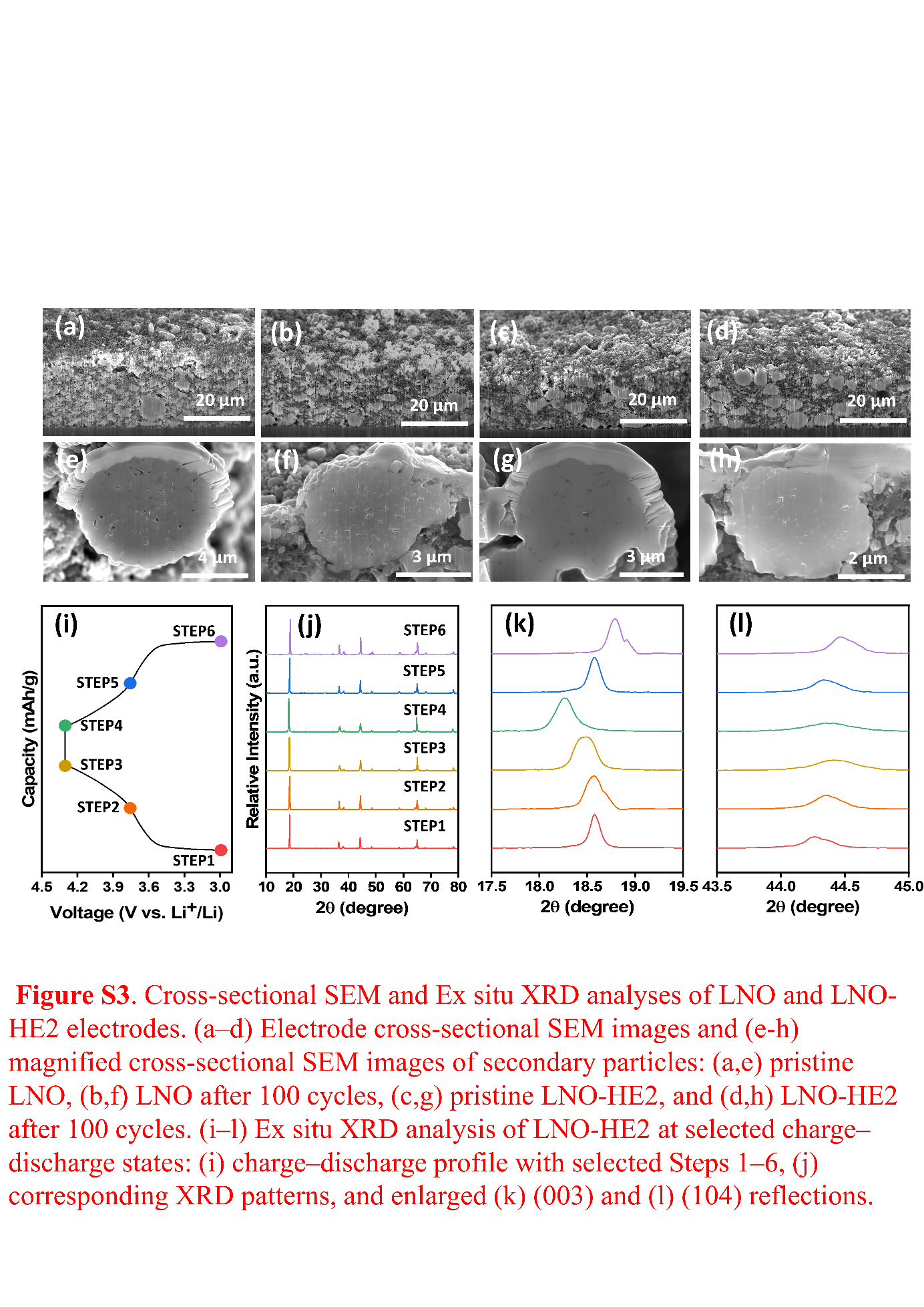


**Figure S4**. Cross-sectional SEM and ex situ XRD analyses of the LNO and LNO-HE2 electrodes. (a–d) Cross-sectional SEM images of the electrode and (e–h) magnified cross-sectional SEM images of secondary particles: (a, e) pristine LNO, (b, f) LNO after 100 cycles, (c, g) pristine LNO-HE2, and (d, h) LNO-HE2 after 100 cycles. (i–l) Ex situ XRD analysis of LNO-HE2 at selected charge–discharge states: (i) charge–discharge profile with selected Steps 1–6, (j) corresponding XRD patterns, and enlarged (k) (003) and (l) (104) reflections.


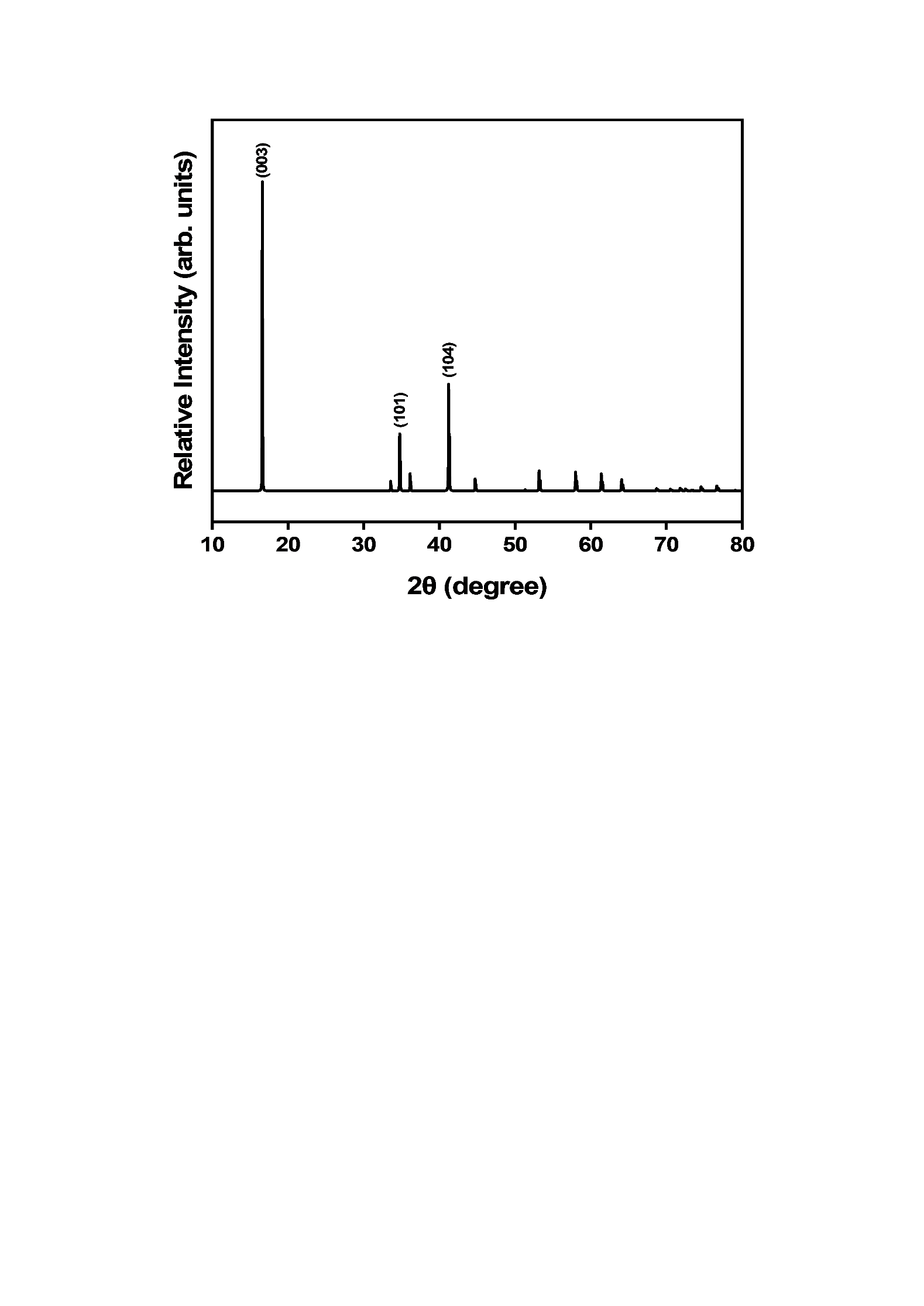


**Figure S5**. Simulated XRD of LNO.


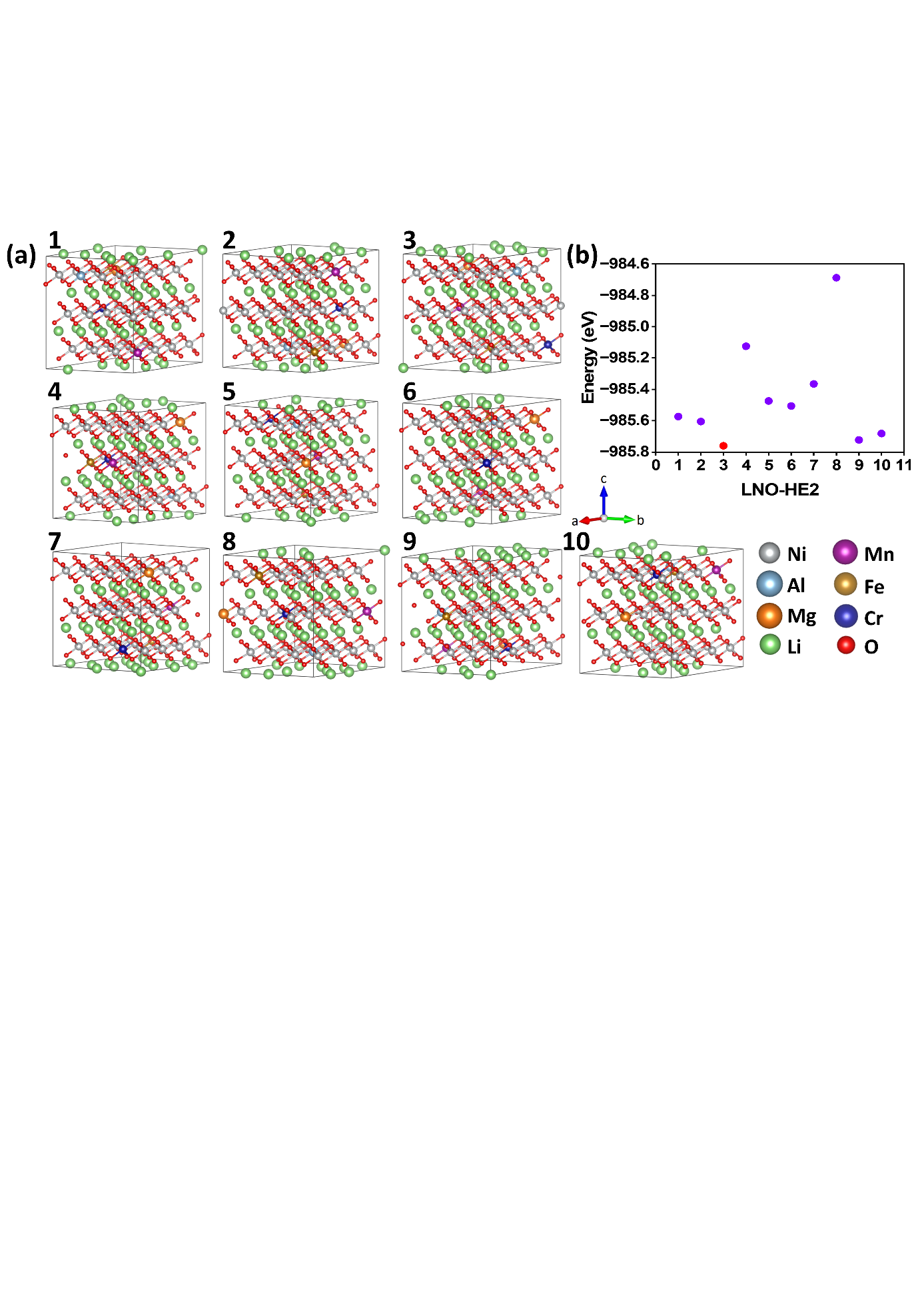


**Figure S6.** (a) Optimized ten high entropy metal oxides and (b) energy correlation of LNO-HE2.


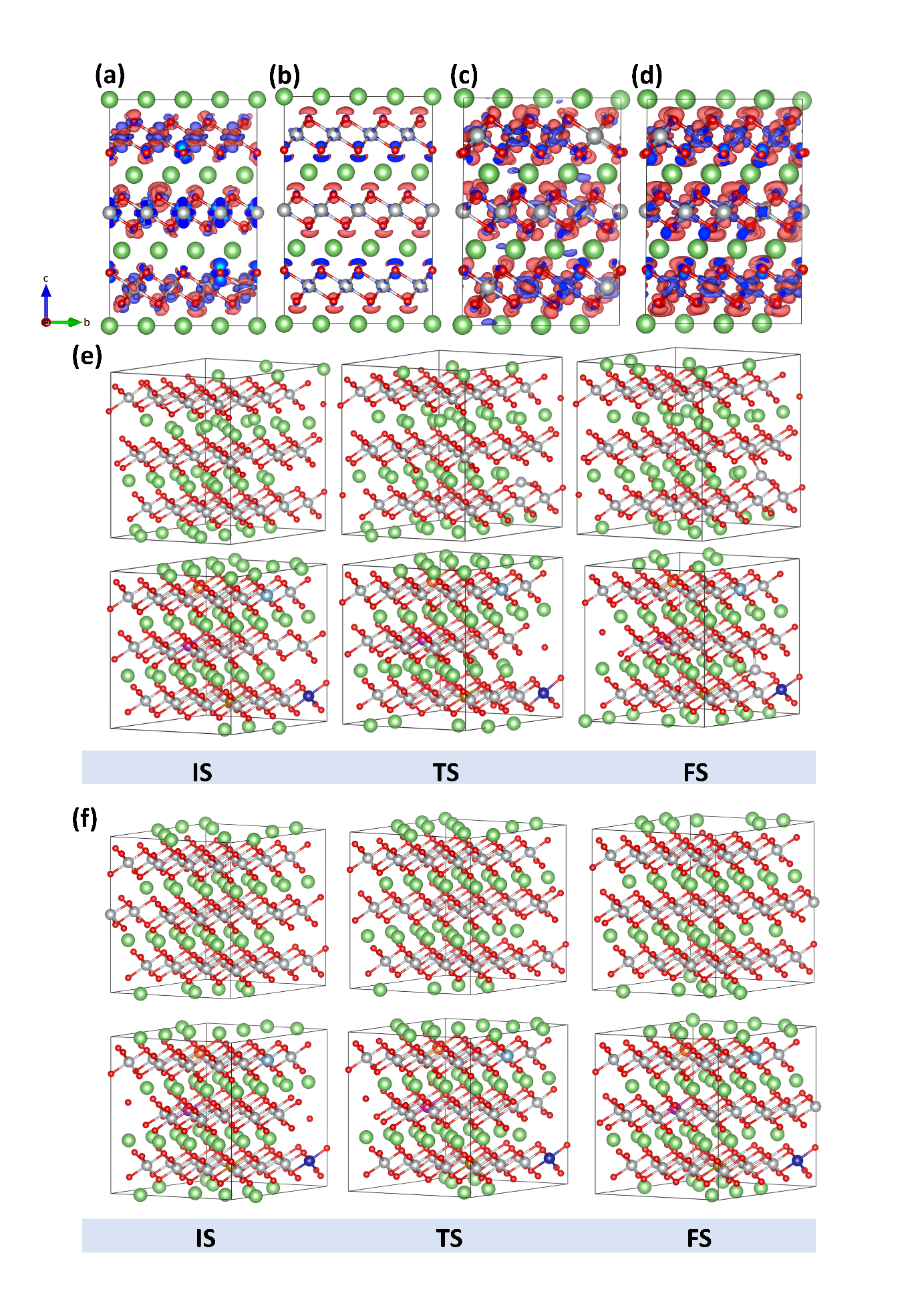


**Figure S7.** (a–d) Charge density difference plots of LNO and LNO-HE2 at half- and full-delithiated states. (e, f) Structural evolution during Ni and Li diffusion in LNO and LNO-HE2 at IS, TS, and FS. Diffused atoms (Ni and Li) are marked with orange circles.
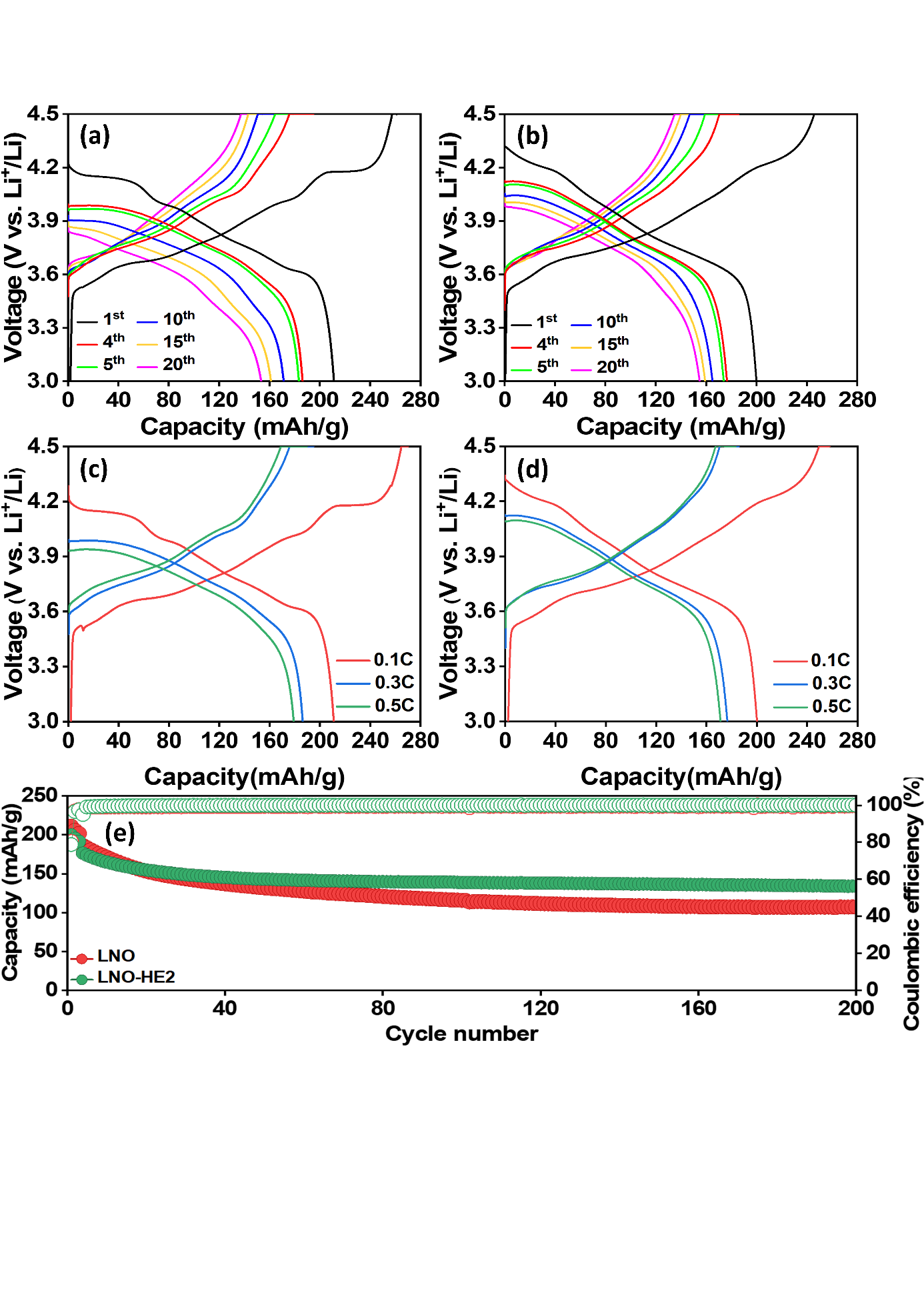
**Figure S8**. Electrochemical performance measured in coin-type full cells assembled with commercial graphite as the anode, showing (a-d) GCD curves at various cycles for (a, c) LNO and (b, d) LNO-HE2. (e) Cycling performance and coulombic efficiency at 0.3C over 200 cycles.

**
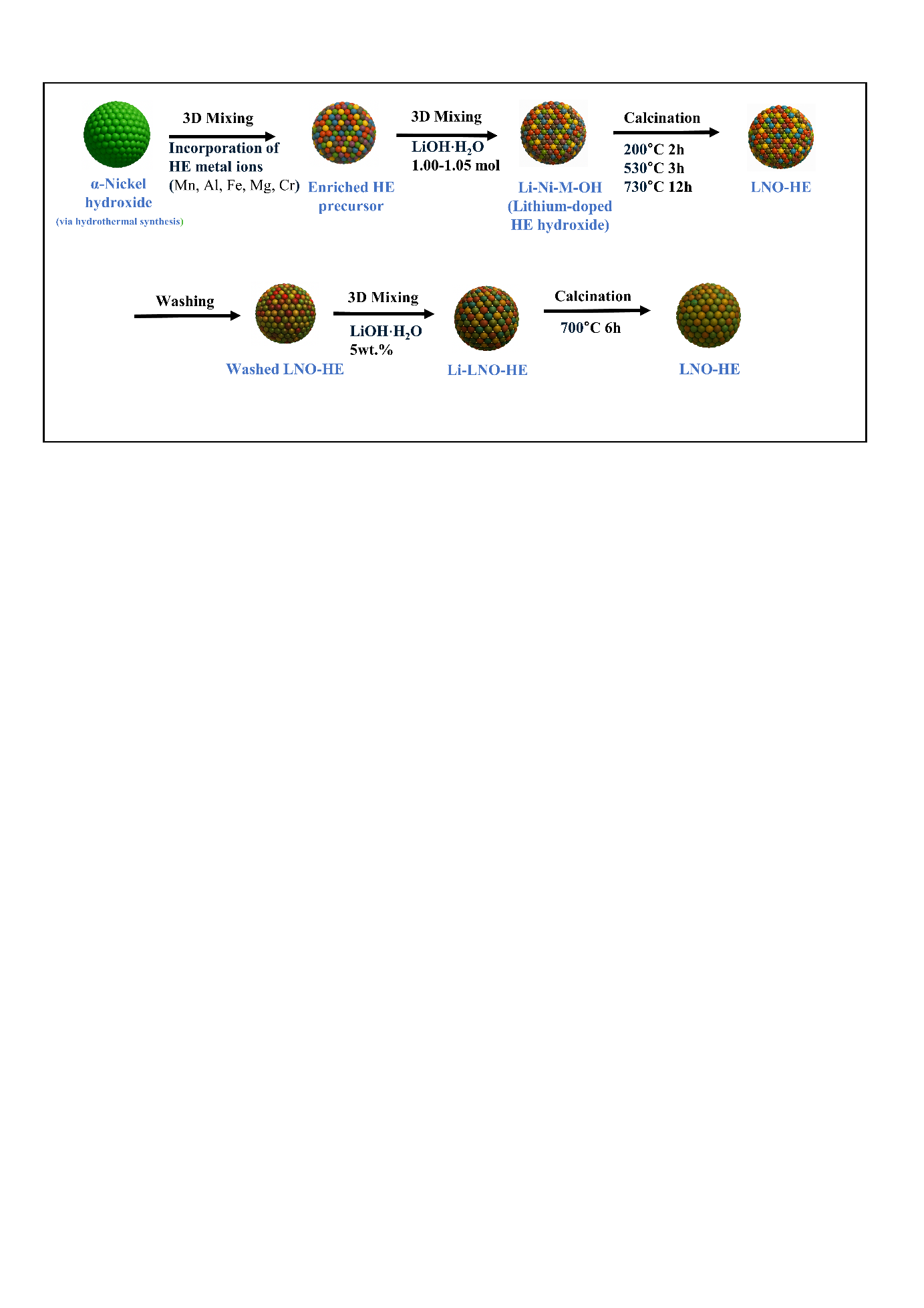
**

**Figure S9.** Diagram of the synthesis procedure for LNO-HE.

**Table S1.** Structural parameters of LNO and LNO-HE cathode materials refined by the Rietveld method from XRD data.

| **Structural parameters** | **LNO** | | **LNO-HE1** | | **LNO-HE2** | | **LNO-HE3** | |  |
| --- | --- | --- | --- | --- | --- | --- | --- | --- | --- |
| a (Å) | 2.87549 | | 2.87554 | | 2.87720 | | 2.87938 | |  |
| b (Å) | 2.87549 | | 2.87554 | | 2.87720 | | 2.87938 | |  |
| c (Å) | 14.19290 | | 14.19824 | | 14.20936 | | 14.22550 | |  |
| Vol. (Å^3^) | 101.631 | | 101.672 | | 101.870 | | 102.140 | |  |
| R_p_ (%) | 2.60 | | 2.74 | | 2.61 | | 2.62 | |  |
| R_wp_ (%) | 3.51 | | 3.80 | | 5.51 | | 3.48 | |  |
| R_I_ (%) | 3.70 | | 4.54 | | 3.93 | | 3.48 | |  |
| R_F_ (%) | 2.73 | | 3.05 | | 2.66 | | 2.99 | |  |
| I_(003)_/I_(104)_ | 1.461 | | 1.877 | | 1.881 | | 1.767 | |  |
| Site | **x** | **Y** | | **z** | | **B_iso_** | | **O_cc_** | |
| **Structural parameters for LNO** | | | | | | | | | |
| Li1 | 0 | 0 | | 0.5 | | 4.04978 | | 0.08150 | |
| Li2 | 0 | 0 | | 0 | | 1.27304 | | 0.00183 | |
| Ni1 | 0 | 0 | | 0.5 | | 4.04978 | | 0.00183 | |
| Ni2 | 0 | 0 | | 0 | | 1.27304 | | 0.08150 | |
| O1 | 0 | 0 | | 0.25792 | | 1.41374 | | 0.16667 | |
| **Structural parameters for LNO-HE1** | | | | | | | | | |
| Li1 | 0 | 0 | | 0.5 | | 2.74485 | | 0.08150 | |
| Li2 | 0 | 0 | | 0 | | 1.18301 | | 0.00183 | |
| Ni1 | 0 | 0 | | 0.5 | | 2.74485 | | 0.00183 | |
| Ni2 | 0 | 0 | | 0 | | 1.18301 | | 0.07760 | |
| Mn1 | 0 | 0 | | 0 | | 1.18301 | | 0.00078 | |
| Al1 | 0 | 0 | | 0 | | 1.18301 | | 0.00078 | |
| Fe1 | 0 | 0 | | 0 | | 1.18301 | | 0.00078 | |
| Mg1 | 0 | 0 | | 0 | | 1.18301 | | 0.00078 | |
| Cr1 | 0 | 0 | | 0 | | 1.18301 | | 0.00078 | |
| O1 | 0 | 0 | | 0.25812 | | 1.35240 | | 0.16667 | |
| **Structural parameters for LNO-HE2** | | | | | | | | | |
| Li1 | 0 | 0 | | 0.5 | | 2.49346 | | 0.08150 | |
| Li2 | 0 | 0 | | 0 | | 1.26424 | | 0.00183 | |
| Ni1 | 0 | 0 | | 0.5 | | 2.49346 | | 0.00183 | |
| Ni2 | 0 | 0 | | 0 | | 1.26424 | | 0.07410 | |
| Mn1 | 0 | 0 | | 0 | | 1.26424 | | 0.00148 | |
| Al1 | 0 | 0 | | 0 | | 1.26424 | | 0.00148 | |
| Fe1 | 0 | 0 | | 0 | | 1.26424 | | 0.00148 | |
| Mg1 | 0 | 0 | | 0 | | 1.26424 | | 0.00148 | |
| Cr1 | 0 | 0 | | 0 | | 1.26424 | | 0.00148 | |
| O1 | 0 | 0 | | 0.25857 | | 1.45817 | | 0.16667 | |
| **Structural parameters for LNO-HE3** | | | | | | | | | |
| Li1 | 0 | 0 | | 0.5 | | 1.33633 | | 0.08150 | |
| Li2 | 0 | 0 | | 0 | | 1.91844 | | 0.00183 | |
| Ni1 | 0 | 0 | | 0.5 | | 1.33633 | | 0.00183 | |
| Ni2 | 0 | 0 | | 0 | | 1.91844 | | 0.07085 | |
| Mn1 | 0 | 0 | | 0 | | 1.91844 | | 0.00213 | |
| Al1 | 0 | 0 | | 0 | | 1.91844 | | 0.00213 | |
| Fe1 | 0 | 0 | | 0 | | 1.91844 | | 0.00213 | |
| Mg1 | 0 | 0 | | 0 | | 1.91844 | | 0.00213 | |
| Cr1 | 0 | 0 | | 0 | | 1.91844 | | 0.00213 | |
| O1 | 0 | 0 | | 0.25624 | | 2.31295 | | 0.16667 | |
| The parameters include lattice constants (a, b, c), unit cell volume, and reliability factors R_p_, R_wp_, R_i_, and R_f_, which represent the goodness of fit for the refinement. Refined atomic coordinates (x, y, z), isotropic displacement parameters (B_iso_), and site occupancies (O_cc_) for LNO and LNO-HE obtained by Rietveld refinement. | | | | | | | | | |

**Table S2.** Comparison of the electrochemical performance of LNO-HE2 and previously reported cathode materials in terms of potential range, initial capacity, and capacity retention.

| Material | Potential range | initial capacity | retention | Ref. |
| --- | --- | --- | --- | --- |
| LNO | 3.0 – 4.5 V | 207.39 mAh g^-1^ (0.1C) | 51 % (100cycles 0.3C) | This work |
| LNO-HE2 | 3.0 – 4.5 V | 204.94 mAh g^-1^ (0.1C) | 78 % (100cycles 0.3C) |  |
| graphite\|\|LNO-HE2 | 3.0 – 4.5 V | 200.13 mAh g^-1^ (0.1C) | 79 % (100cycles 0.3C) |  |
| Hydrothermal LNO | 3.0 – 4.3 V | 211.9 mAh g^-1^ (0.1C) | 77% (50cycles 0.5C) | [10] |
| C-precipitation LNO | 3.0 – 4.3 V | 163.6 mAh g^-1^ (0.1C) | 66% (50cycles 0.5C) | [10] |
| Graphene coated LNO | 2.8 – 4.6 V | ~185 mAh g^-1^ (1C) | 76% (100cycles 1C) | [11] |
| Y based oxides coated LNO | 2.8 – 4.3 V | 225 mAh g^-1^ (0.1C) | 63% (100cycles 0.5C) | [12] |
| Ga doped LNO | 3.0 – 4.3 V | 174 mAh g^-1^ (0.1C) | 76% (100cycles 0.3C) | [13] |
| Mo doped LNO | 2.7 – 4.3 V | 240 mAh g^-1^ (10mA g^-1^) | 52% (100cycles, 10mA g^-1^) | [14] |
| Na substituted LNO | 3.0 – 4.4 V | 192 mAh g^-1^ (0.1C) | 76% (100cycles 0.5C) | [15] |
| TNO\|\|F doped LNO | 1.5 – 3.5 V | 175 mAh g^-1^ (0.2C) | 65% (100cycles 0.2C) | [16] |

**Table S3.** Voltage-dependent Li^+^ diffusion coefficients (D) of LNO and LNO-HE2 estimated from the low-frequency Warburg region of in situ EIS during charge (3.0–4.5 V) and discharge (4.4–3.0 V).

| **Process** | **Voltage (V)** | **LNO (cm² s⁻¹)** | **LNO-HE2 (cm² s⁻¹)** |
| --- | --- | --- | --- |
| Charging | 3.0 | 1.80 × 10⁻¹³ | 2.47 × 10⁻¹³ |
|  | 3.2 | 2.29 × 10⁻¹³ | 2.98 × 10⁻¹³ |
|  | 3.4 | 2.71 × 10⁻¹³ | 3.39 × 10⁻¹³ |
|  | 3.6 | 1.97 × 10⁻¹³ | 3.47 × 10⁻¹² |
|  | 3.8 | 2.82 × 10⁻¹⁰ | 1.74 × 10⁻¹⁰ |
|  | 4.0 | 3.00 × 10⁻¹⁰ | 3.05 × 10⁻¹⁰ |
|  | 4.2 | 9.02 × 10⁻¹¹ | 3.09 × 10⁻¹⁰ |
|  | 4.4 | 9.92 × 10⁻¹³ | 1.08 × 10⁻¹¹ |
|  | 4.5 | 2.28 × 10⁻¹³ | 1.15 × 10⁻¹² |
| Discharging | 4.4 | 1.65 × 10⁻¹³ | 6.49 × 10⁻¹³ |
|  | 4.2 | 1.67 × 10⁻¹² | 3.77 × 10⁻¹⁰ |
|  | 4.0 | 2.27 × 10⁻¹⁰ | 2.90 × 10⁻¹⁰ |
|  | 3.8 | 1.58 × 10⁻¹⁰ | 2.05 × 10⁻¹⁰ |
|  | 3.6 | 8.51 × 10⁻¹¹ | 3.25 × 10⁻¹¹ |
|  | 3.4 | 3.09 × 10⁻¹³ | 3.84 × 10⁻¹³ |
|  | 3.2 | 1.44 × 10⁻¹³ | 2.33 × 10⁻¹³ |
|  | 3.0 | 1.69 × 10⁻¹³ | 2.38 × 10⁻¹³ |

**References**

[1] G. Kresse, J. Furthmüller, “Efficient Iterative Schemes for *Ab Initio* Total-Energy Calculations Using a Plane-Wave Basis Set,” *Physical Review B* 54 (1996): 11169–11186. <https://doi.org/10.1103/PhysRevB.54.11169>

[2] G. Kresse, J. Hafner, “*Ab Initio* Molecular-Dynamics Simulation of the Liquid-Metal–Amorphous-Semiconductor Transition in Germanium,” *Physical Review B* 49 (1994): 14251–14269. <https://doi.org/10.1103/PhysRevB.49.14251>

[3] G. Kresse, J. Hafner, “*Ab Initio* Molecular Dynamics for Liquid Metals,” *Physical Review B* 47 (1993): 558–561. <https://doi.org/10.1103/PhysRevB.47.558>

[4] G. Kresse, J. Furthmüller, “Efficiency of Ab-Initio Total Energy Calculations for Metals and Semiconductors Using a Plane-Wave Basis Set,” *Computational Materials Science* 6 (1996): 15–50. <https://doi.org/10.1016/0927-0256(96)00008-0>

[5] P. E. Blöchl, “Projector Augmented-Wave Method,” *Physical Review B* 50 (1994): 17953–17979. <https://doi.org/10.1103/PhysRevB.50.17953>

[6] G. Kresse, D. Joubert, “From Ultrasoft Pseudopotentials to the Projector Augmented-Wave Method,” *Physical Review B* 59 (1999): 1758–1775. <https://doi.org/10.1103/PhysRevB.59.1758>

[7] J. P. Perdew, K. Burke, and M. Ernzerhof, “Generalized Gradient Approximation Made Simple,” *Physical Review Letters* 77 (1996): 3865–3868. <https://doi.org/10.1103/PhysRevLett.77.3865>

[8] S. L. Dudarev, G. A. Botton, S. Y. Savrasov, C. J. Humphreys, and A. P. Sutton, “Electron-Energy-Loss Spectra and the Structural Stability of Nickel Oxide: An LSDA+U Study,” *Physical Review B* 57 (1998): 1505–1509. <https://doi.org/10.1103/PhysRevB.57.1505>

[9] H. J. Monkhorst, J. D. Pack, “Special Points for Brillouin-Zone Integrations,” *Physical Review B* 13 (1976): 5188–5192. <https://doi.org/10.1103/PhysRevB.13.5188>

[10] [T.](https://doi.org/10.1016/j.matlet.2022.131810) T. B. Tran, E.-J. Park, H.-I. Kim, S.-H. Lee, H.-J. Jang, and J.-Tae Son, “High Rate Performance of Lithium-Ion Batteries with Co-Free LiNiO_2_ Cathode,” *Materials Letters* 316 (2022): 131810. <https://doi.org/10.1016/j.matlet.2022.131810>

[11] [K.](https://doi.org/10.1002/adma.202106402)-Y. Park, Y. Zhu, C. G. T.-Castanedo, H. J. Jung, N. S. Luu, O. Kahvecioglu, Y. Yoo, J.-W. T. Seo, J. R. Downing, H.-D. Lim, M. J. Bedzyk, C. Wolverton, and M. C. Hersam, “Elucidating and Mitigating High‐Voltage Degradation Cascades in Cobalt‐Free LiNiO_2_ Lithium‐Ion Battery Cathodes,” *Advanced Materials* 34 (2022): 2106402. <https://doi.org/10.1002/adma.202106402>

[12] [Y.](https://doi.org/10.1016/j.jechem.2021.07.029) Zhang, H. Li, J. Liu, J. Liu, H. Ma, and F. Cheng, “Enhancing LiNiO_2_ Cathode Materials by Concentration-Gradient Yttrium Modification for Rechargeable Lithium-Ion Batteries,” *Journal of Energy Chemistry* 63 (December 2021): 312–319. <https://doi.org/10.1016/j.jechem.2021.07.029>

[13] M. Mishra, K. P. C. Yao, “The Emergence of a Robust Lithium Gallium Oxide Surface Layer on Gallium-Doped LiNiO_2_ Cathodes Enables Extended Cycling Stability,” *Materials Advances* 5 (2024): 7016–7027. <https://doi.org/10.1039/D3MA01102J>

[14] [J.-M.](https://doi.org/10.1039/D3YA00046J) Price, P. Allan, and P. Slater, “Optimising the Synthesis of LiNiO_2_: Coprecipitation *versus* Solid-State, and the Effect of Molybdenum Doping,” *Energy Advances* 2 (2023): 864–876. <https://doi.org/10.1039/D3YA00046J>

[15] H. Kim, A. Choi, S. W. Doo, J. Lim, Y. Kim, and K. T. Lee, “Role of Na^+^ in the Cation Disorder of [Li_1-x_Na_x_]NiO_2_ as a Cathode for Lithium-Ion Batteries,” *Journal of The Electrochemical Society* 165 (2018): A201–A205. <https://doi.org/10.1149/2.0771802jes>

[16] [E.](https://doi.org/10.1002/cssc.202500300) ELmaataouy, M. E. Kassaoui, M. Elmouhinni, K. Kubota, A. Chari, M. Aqil, A.Sghiouri, J. Alami, O. Mounkachi, and M. Dahbi, “Effect of Fluorine Doping on the Electrochemistry and Structural Stability of Single‐Particle LiNiO_2_,” *ChemSusChem* 18 (2025): e202500300. <https://doi.org/10.1002/cssc.202500300>
